# Challenges and Advances in Biomarker Detection for Rapid and Accurate Sepsis Diagnosis: An Electrochemical Approach

**DOI:** 10.3390/bios14060309

**Published:** 2024-06-17

**Authors:** Deivasigamani Ranjith Kumar, Angelika Banaś, Katarzyna Krukiewicz

**Affiliations:** 1 Centre for Organic and Nanohybrid Electronics, Silesian University of Technology, Konarskiego 22B, 44-100 Gliwice, Poland; ranjith.kumar.deivasigamani@polsl.pl; 2 Department of Physical Chemistry and Technology of Polymers, Silesian University of Technology, M. Strzody 9, 44-100 Gliwice, Poland; angeban429@student.polsl.pl

**Keywords:** sepsis, electrochemical sensor, blood sample, biomarker detection

## Abstract

Sepsis is a life-threatening condition with high mortality rates due to delayed treatment of patients. The conventional methodology for blood diagnosis takes several hours, which suspends treatment, limits early drug administration, and affects the patient’s recovery. Thus, rapid, accurate, bedside (onsite), economical, and reliable sepsis biomarker reading of the clinical sample is an emergent need for patient lifesaving. Electrochemical label-free biosensors are specific and rapid devices that are able to perform analysis at the patient’s bedside; thus, they are considered an attractive methodology in a clinical setting. To reveal their full diagnostic potential, electrode architecture strategies of fabrication are highly desirable, particularly those able to preserve specific antibody–antigen attraction, restrict non-specific adsorption, and exhibit high sensitivity with a low detection limit for a target biomarker. The aim of this review is to provide state-of-the-art methodologies allowing the fabrication of ultrasensitive and highly selective electrochemical sensors for sepsis biomarkers. This review focuses on different methods of label-free biomarker sensors and discusses their advantages and disadvantages. Then, it highlights effective ways of avoiding false results and the role of molecular labels and functionalization. Recent literature on electrode materials and antibody grafting strategies is discussed, and the most efficient methodology for overcoming the non-specific attraction issues is listed. Finally, we discuss the existing electrode architecture for specific biomarker readers and promising tactics for achieving quick and low detection limits for sepsis biomarkers.

## 1. Introduction

Sepsis is a life-threatening condition caused by the exaggerated response of the immune system as a result of infection [1]. When left untreated, sepsis can lead to serious disorders of organ functions and death. Normally, when the body comes into contact with a pathogen, it initiates an immune response to control the infection, but in the case of sepsis, this response becomes excessive, leading to a widespread inflammatory state [2]. According to the World Health Organization (WHO), sepsis causes around 11 million deaths annually and is one of the most common causes of death worldwide [3]. There is no clear correlation between the age of patients and the incidence of sepsis [4]; however, its incidence is related to wealth, with low-income countries being the most affected [5,6]. If not treated in time, sepsis, even when not fatal, can cause irreversible side effects and complications such as chronic weakness, sleep problems, weight loss, hair loss, and worsening skin conditions [5]. Mental health impacts should not be overlooked, as patients often feel isolated, suffer from anxiety disorders, and exhibit attention deficit disorders and depression. As indicated by several research studies, many sepsis patients exhibit post-traumatic stress disorder [7,8] and sepsis-associated dysfunctions [9]. For many people, surviving sepsis is associated with regular returns to the hospital and the need to be under constant medical supervision, which significantly affects their quality of life [9]. 

The development of sepsis in the body can be divided into three main stages: early sepsis, severe sepsis, and septic shock [10,11]. The first stage of sepsis can last from a few hours to even a few days and causes nonspecific symptoms such as fever, weakness, accelerated heart rate, or breathing problems [12]. These symptoms can develop suddenly and may initially be mild or resemble other conditions, making early detection of sepsis difficult. Within hours to a few days, these symptoms can escalate rapidly. Early intervention at this stage can significantly improve patient outcomes, since the next stage of sepsis (i.e., severe sepsis) manifests itself in organ dysfunction, difficulty breathing, reduced urine output, increased heart rate, and elevated blood pressure. The risk of mortality increases substantially when sepsis progresses to this stage [10,13]. Even after just a few hours, severe sepsis can develop into septic shock, which is identified by a significant drop in blood pressure that does not respond adequately to fluid replacement, along with signs of organ failure [10,14]. This stage is associated with the highest mortality rate, and treatment must be given immediately. 

## 2. Need for a Rapid Sepsis Sensor

Sepsis is one of the major causes of in-hospital mortality in intensive care unit (ICU) patients worldwide. When the patient is admitted to the ICU, treatment needs to start within two to three hours (the “golden hour”). During this time, whole blood monitoring is highly desirable for patient lifesaving. Angus et al. [15] estimated 751,000 sepsis cases per year were recorded in the USA, and among them, 28.6% resulted in the death of the patient. United Kingdom reports say that 100,000 sepsis patients are admitted to ICUs each year, with a mortality rate of 35%, which is higher than for colon, breast, and lung cancer patients [16]. The National Health Care system in Poland reports the percentages of sepsis patients admitted to ICUs daily as 26% (2012) and 22% (2013) [17]. Delayed sepsis diagnosis increases the chance of mortality by 6% to 10% per hour [18]. Therefore, it is essential to speed up the treatment time for sepsis as much as possible, and this is linked to the need to develop solutions for the rapid detection of progressive symptoms in the body. 

Unfortunately, traditional methods of detecting sepsis fail in many cases. Traditional methods of sepsis sensing are based on blood cultures, where it takes around 24 h to obtain results, which could be too long because the timing of detection is crucial in sepsis treatment. For example, it takes 14–48 h to perform basic analysis of a patient’s blood to identify bacterial pathogens, which is well beyond the usual time taken for sepsis to develop to its worst stage [19]. Although polymerase chain reaction (PCR) testing appears to be a precise and fast diagnostic method, it is important to remember that it involves several steps, such as the collection of blood samples and the interpretation of results. Thus, it can be said that a properly performed procedure can take approximately several hours. Faster methods, including complete blood counts (CBCs), imaging tests, and clinical assessments, are usually not specific enough to indicate sepsis. In light of the above, it is clear that there is a need to develop an appropriate method of detecting sepsis, enabling treatment to be initiated early in the disease’s development when the risk of death is lowest [19,20]. In 80% of cases, death from sepsis can be prevented if the condition is detected in time and adequately treated. It is assumed that the best time to react is within one hour after the first symptoms of sepsis appear, which is challenging due to their low specificity. The development of an appropriate detection system will not only increase patient survival rates but may also contribute to reducing the economic costs associated with the difficult and costly treatment of patients who survive septic shock and are struggling with post-septic symptoms [21]. That is why the time of diagnosis should be as short as possible [22]. 

## 3. Sepsis Blood Sample Handling

**Figure 1:**
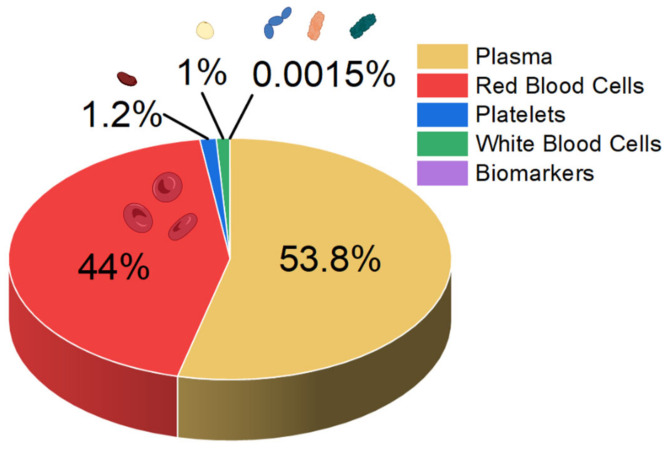
Approximate composition of the blood of a person with sepsis.

Sepsis blood differs significantly in composition from blood from a healthy person. In the early stage of sepsis, the number of white blood cells (WBCs) can increase, mainly owing to the immune response of the body. In a normal person, the WBC count is typically less than 11,000, but in early sepsis patients it doubles, and in the late stage it drastically decreases [23]. Also, neutrophilia-to-lymphocyte ratio values increase to 23.8 [24,25]. Furthermore, the sepsis patient’s biomarker concentrations exceed 100 mg L^−1^ and 10 ng mL^−1^ for C-reactive protein and procalcitonin, respectively [23]. While normal platelet counts in blood range from 150,000 to 450,000 platelets per μL, they can decrease with sepsis, as platelets are consumed during the formation of microclots in the body’s attempt to isolate the infection (thrombocytopenia) [25,26]. A schematic summary of the blood composition of a septic patient is shown in Figure 1. 

**Figure 2:**
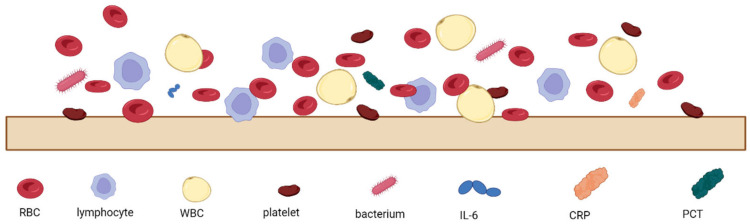
Schematic representation of molecular crowding of blood components on the sensor’s surface, where RBC—red blood cells, WBC—white blood cells, IL-6—interleukin-6, CRP—C-reactive protein, and PCT—procalcitonin.

The analysis of patients’ blood is the most common form of sepsis diagnosis. However, it has its drawbacks, the main one being the time needed for the analysis, which is due to the special procedures for handling blood samples [27]. When looking at the blood composition of a person with sepsis (Figure 1), it can be seen that sepsis biomarkers make up a small proportion of the blood (0.0015%). It is important to bear in mind that using a blood sample directly on the sensor’s surface will cause all the components of the blood to interact with the sensor, which can distort and slow down the measurement. Figure 2 schematically shows the molecular crowding of various blood components on the sensor’s surface, highlighting the need to design a sensing material that is able to interact selectively with desired biomarkers and is able to resist non-specific interactions with other blood components.

In addition to dysregulated amounts of blood components (WBCs, CRP, PTC, and platelets), sepsis blood may also contain microorganisms. Sepsis can be caused by a wide variety of bacterial strains, which can enter the bloodstream and trigger a systemic inflammatory response. The most common bacterial strains that can lead to sepsis include both Gram-positive bacteria (*Staphylococcus epidermidis*, *Staphylococcus aureus*, *Streptococcus mitis*, and *Enterococcus species*) and Gram-negative bacteria (*Escherichia coli*, *Pseudomonas aeruginosa*, and *Klebsiella species*) [28,29], as well as some types of fungi (*Candida albicans*, *Ascomycota*, and *Basidiomycota*) [30]. Therefore, special care must be taken when analyzing a patient’s blood. First and foremost, tests should be carried out by following standard microbiological practices with the use of a certified Class II Biological Safety Cabinet (BSC). Personnel should wear appropriate Personal Protective Equipment (PPE), which includes gloves, lab coats, glasses, and safety masks. Samples should be clearly labeled as potentially infectious and stored in sealed, leak-proof containers to prevent accidental exposure during transportation. All working areas should be disinfected before and after processing with the use of hospital-grade disinfectant. Waste material and used PPE should be disposed of in biohazard bags with consideration of sharps, which should be in puncture-resistant containers. Staff must be properly trained and adhere to guidelines on the handling of infectious materials, mainly from the Centers for Disease Control and Prevention (CDC) and WHO [31,32].

**Figure 3:**
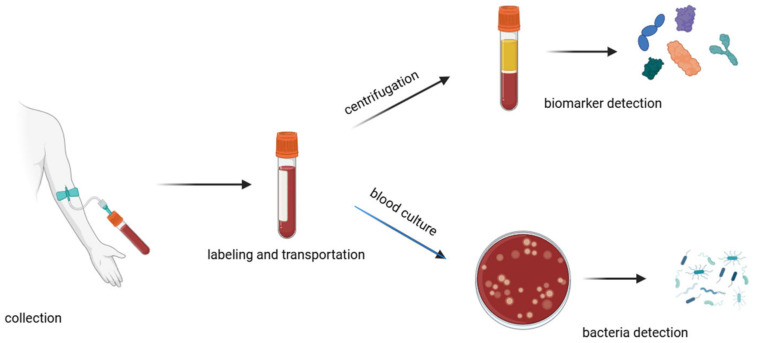
Schematic of the blood handling procedure in hospitals.

A traditional method for diagnosing sepsis is to analyze the patient’s blood composition (Figure 3). This is a time-consuming method that can be divided into major steps: collection, labeling, transportation, processing, testing, and documentation [33]. The collection of samples has to be carried out with strict aseptic techniques to avoid contamination of the blood sample. It is important to draw an adequate volume of blood (approximately 20–30 mL) [34] so that all the necessary tests can be performed. Unfortunately, in the case of sepsis, when it is necessary to monitor changes in blood composition at intervals, it is not possible to draw such large volumes of blood so often, as this can significantly weaken the patient’s health and in some cases even endanger the patient’s life. The transportation process should be as quick as possible because delays can affect the viability of pathogens and the integrity of biomarkers. During the transport of the sample, the temperature needs to be controlled, and any physical damage needs to be avoided to prevent leakage, shock, or vibration. There is also a need to avoid direct sunlight, which can alter the stability of certain analytes or lead to hemolysis [35]. Inadequate sample storage can also affect the stability of sepsis biomarkers (e.g., IL-6), which can significantly interfere with appropriate diagnosis [36]. Upon arrival in the laboratory, the blood sample is prepared for analysis by aliquoting the blood samples into appropriate containers, storing them with nutrient media, and incubating. 

Often, incubators are equipped with tools and programs to monitor the growth of microorganisms; e.g., with colorimetric indicators or pressure sensing [37]. In cases of the detection of bacteria growth, personnel are alerted and undertake further steps to isolate and identify the pathogens using microscopy, biochemical tests, or molecular biology methods [27,28]. For biomarker assays, blood samples should be first centrifugated to separate plasma from the blood cells [33]. For further tests, such as Enzyme-Linked Immunosorbent Assay (ELISA), chemiluminescence immunoassays, or electrochemiluminescence assays, the sample is mixed with specific reagents that cause a detectable reaction (such as a color change or luminescence) in the presence of the target biomarker [19,27]. This involves a specific reaction between the antibodies and targeted biomarkers. A secondary antibody that is linked to an enzyme or fluorescent label binds to the complex, enabling detection.

In some cases, removing inflammatory mediators and toxins from the blood is used to improve outcomes in septic patients. This can be achieved using various blood purification techniques, such as hemofiltration, hemodiafiltration, hemoperfusion, plasma filtration, and adsorption. These therapies help in clearing harmful substances from the blood, leading to improvements in various physiological outcomes such as hemodynamics and oxygenation. The removal of these inflammatory mediators or bacterial products helps in reducing the systemic inflammatory response associated with sepsis, potentially leading to better clinical outcomes for patients. However, these techniques can lead to anticoagulation and potential device-related complications. There is also a lack of sufficient large-scale clinical trials to definitively establish their efficacy in improving clinical outcomes in patients with sepsis [38,39]. 

## 4. Sepsis Biomarkers

**Figure 4:**
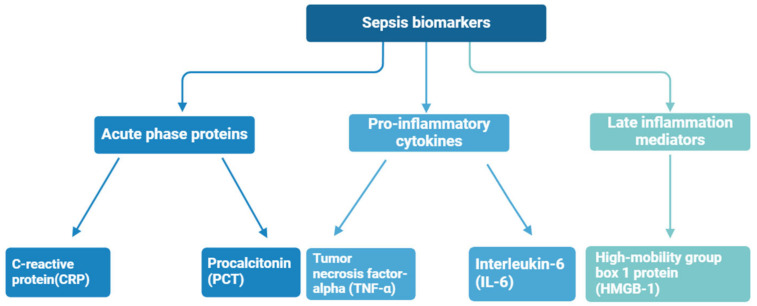
Sepsis biomarkers divided by immune response phase.

Biomarkers are biological compounds that are used as a signature of physiological or pathological conditions and can be found in clinical samples of whole blood, serum, plasma, and cellular fluid. To date, numerous sepsis monitoring biomarkers have been investigated, with a total of more than 100 kinds of sepsis biomarkers identified at different stages of sepsis [40]—the most important of them are shown in Figure 4. Sepsis diagnosis is based on the presence of certain biomarkers in the patient’s blood, for instance, C-reactive protein (CRP), procalcitonin (PCT), tumor necrosis factor-alpha (TNF-α), high-mobility group box 1 protein (HMGB-1), and interleukin-6 (IL-6) [41,42]. These biomarkers are often associated with an inflammatory state, but significantly elevated concentrations may indicate an increased immune response and the presence of sepsis. 

CRP is secreted primarily in the kidney, liver, and atherosclerotic tissues and travels through the plasma [43]. CRP is an acute-phase protein; it is a signature of cardiovascular diseases and related infections. The average CRP level in a healthy person is 2 mg mL^−1^, and for sepsis patients it increases more than 1000 times [44]. However, its levels can also rise in other conditions, such as fungal and bacterial infections, inflammatory conditions, and also following surgery, meaning it is not a specific sepsis biomarker [41]. In recent studies, CRP has also been explored as a potential biomarker for other diseases, like COVID-19 [45], Alzheimer’s disease [46], and rheumatoid arthritis [47]. 

Calcitonin precursor (procalcitonin, PCT) was discovered in 1975 by Moya et al. [48]. PCT plays a role in phosphate and calcium metabolism (calcitonin prohormone). Consisting of a combination of 116 amino acids, PCT is created in thyroid C cells. A normal person’s PCT level is >1 ng mL^−1^; in contrast, for sepsis patients, an elevated level (>1 ng mL^−1^) is observed [43]. After the onset of sepsis, PCT levels drastically increase within 2 days [41,49]. Therefore, regular monitoring of PCT levels increases patient survival rates and reduces doctors’ burden. 

TNF-α was identified in 1975 by William B Coley, who was the first to find out that bacteria produce an endotoxin that is able to provoke tumor necrosis [50,51]. TNF-α is a pro-inflammatory cytokine with a molecular weight of 17.5 kDa that activates the acute-phase reaction. TNF-α plays a key role in the body’s immune response by regulating immune cells, inducing fever, causing apoptotic cell death, sepsis, and inflammation, and inhibiting tumorigenesis and viral replication. Dysregulation of the production of TNF-α has been linked to various diseases, including autoimmune diseases, insulin resistance, and cancer [52]. Under normal conditions, TNF-α levels are <6 ng mL^−1^ and increase under infected conditions [43]. A high concentration of TNF-α is an indicator of heart disease, cancer, autoimmune diseases, and diabetes. 

IL-6, which was first identified by Kishimoto’s research group [53], is a 26 kDa soluble protein liberated by T cells, which stimulate B cells to secrete antibodies. Initially called B cell stimulatory factor 2 (BSF-2), IL-6 has been also known as IFN-β2, BSF-2, and hepatocyte stimulating factor [54,55]. IL-6 is a kind of glycoprotein produced by lymphocytes and macrophages; a healthy level is 15 pg mL^−1^, increasing to over 3 ng mL^−1^ for a sepsis patient [56]. IL-6 is a cytokine involved in the body’s immune responses associated with inflammation. It is a key mediator of fever and inflammatory response and has been implicated in the pathogenesis of many diseases, including autoimmune diseases, sepsis, and cancer. IL-6 acts as both a pro-inflammatory cytokine and an anti-inflammatory myokine, indicating its complex role in modulating the immune response [42]. 

HMGB1 is a nuclear protein that plays an important role in regulating gene expression by binding to DNA. It also acts as a pro-inflammatory cytokine when released extracellularly and is associated with the pathogenesis of various inflammatory and autoimmune diseases. HMGB1 may contribute to the persistence of inflammatory responses, making it a target for therapeutic intervention in conditions characterized by chronic inflammation, as seen in sepsis [57].

Quantification of sepsis biomarkers can greatly facilitate diagnosis [58,59]. Even though the detection of some of them (CRP and PCT, for instance) can be fast, the acquired results lack specificity, as their levels can also rise in non-septic conditions [58]. It should also be borne in mind that in the case of sepsis, a sharp and progressive increase in the concentration of the biomarkers mentioned (e.g., IL-6) can be observed, whereas in the case of other diseases, for instance ovarian cancer, their concentration is elevated but does not progress significantly over time [60,61]. Sensors capable of the simultaneous detection of several biomarkers of sepsis are the solution here. They not only enable efficient and rapid detection of sepsis but may also aid in the treatment process by indicating whether it is successful or should be changed. The quantification of specific biomarkers in the presence of a mixture of other biomarkers is crucial, and success depends on the specificity and sensitivity of a respective sensor probe. Taking all these constraints and requirements into account, the use of electrochemical sensors emerges as the best solution. First and foremost, they enable rapid detection, while maintaining high sensitivity and selectivity [62]. The combination of these features and the possibility of repeated measurements allows for the monitoring of subtle changes in biomarker concentrations over time, thus decreasing the diagnosis time and increasing the efficiency of the treatment.

## 5. Types of Sepsis Sensor 

Owing to the need for rapid and accurate sepsis detection methods, modern science proposes various solutions for the analysis of sepsis biomarkers. Sensors for the diagnosis of sepsis include the following: (1) optical, (2) field effect transistor (FET), (3) microfluidic, and (4) electrochemical sensors [63]. This section will describe these detection methods briefly and compare them with each other.

### 5.1. Optical Sensors 

Optical sensors detect changes in light properties (such as absorbance, fluorescence, or scattering) in response to microbial growth or the presence of specific biomarkers. The most promising in terms of actual use in hospitals appears to be fluorescent sensors. Fluorescence-based sensors exploit the property of certain molecules to absorb light at a specific wavelength and then emit light at a longer wavelength. Here, two main techniques can be described, namely fluorescent labeling, where sepsis-related biomarkers are tagged with fluorescent dyes, and fluorescent in situ hybridization (FISH), where fluorescent probes bind to specific DNA sequences of sepsis-related microbes [63]. For example, a system using nanoclusters to detect bacteria associated with sepsis was proposed as a potential point-of-care device for the detection of sepsis in children, since it could reduce the need for blood cultures, thereby greatly improving diagnosis [64]. The detection time here was very short (15 s), and the sensor itself showed high sensitivity and linearity of measurement, where the detection limits for the *S. aureus*, *E. coli*, *P. aeruginosa* bacterial strains were respectively 43, 26, and 47 CFU mL^−1^. Another example of an optic sepsis sensor is based on surface-enhanced Raman spectroscopy (SERS), allowing for the identification of nucleic acid sequences associated with bacterial infections leading to sepsis. Those devices enable accurate readings to facilitate early diagnosis and treatment of sepsis, potentially improving patient outcomes [58]. Unfortunately, Raman spectroscopy is not a workable solution for use in hospitals. Optical sensors provide high sensitivity and ease to fluorescent probes with reader unit functionalization or colorimetric response reactions [65]. However, the optical sensor’s major limitation is its poor resolution with volume of the target analyte [66]. First of all, it takes a long time, which is extremely important for the diagnosis of sepsis, in addition to the fact that the equipment itself takes up a lot of space, is not easy to operate, and requires skilled staff. A summary of the pros and cons of optical sensors is presented in Table 1. 

### 5.2. Field-Effect Transistor (FET) Sensors

FET sensors measure changes in electrical conductivity as a result of biomolecular interactions on their surface, which affect the current flow through the transistor. A typical FET sensor works by testing the conductivity between two elements, source and drain, when an electric field is applied to the so-called gate. Modern science offers solutions in which this gate is modified with biological elements capable of binding to a specific biomarker. The formation of bonds between the biomarker and the elements on the surface causes a change in the electric field, which reduces the current flowing through the transistor. An interesting solution was proposed by Chen et al. [72], where the graphene sensor was modified with chitosan to ensure high selectivity and stability, significantly improving the sensor’s performance to achieve ultra-sensitive detection of PCT (LOD = 0.82 ag mL^−1^). A definite advantage of this type of sensor is its ability to be miniaturized and used as a point-of-care device. Unfortunately, many FET sensors still require pre-treatment of the blood, where testing is only carried out with blood plasma [73].

### 5.3. Electrochemical Sensors 

**Figure 5:**
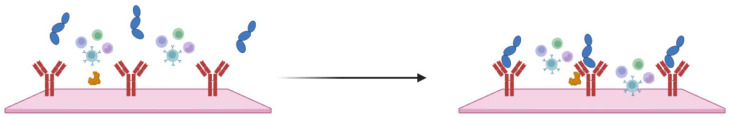
Schematic representation of IL-6 binding to an antibody, causing a change in system impedance.

Electrochemical sensors detect changes in electrical properties (such as voltage, current, or impedance) in response to the presence of specific biomolecules or pathogens. Specific receptors or antibodies are placed on the surface of the working electrode, which can selectively bind to sepsis biomarkers such as specific proteins, pathogen DNA, toxins, or other molecules indicative of the inflammatory process. When the biomarkers present in the blood sample bind to the receptors, there is a change in the electrical properties of the electrode surface (Figure 5) [74].

A detailed overview and comparison of electrochemical sensors will be described in the following sections. Nevertheless, it is already possible to list the advantages of this type of sensor, which appears to be the best choice for use in the diagnosis of sepsis. Although optical sensors show high selectivity and sensitivity, they are very expensive. Electrochemical sensors are also sensitive and selective, yet their cost is significantly less, making them suitable for use in hospitals. Electrochemical sensors can be also designed to be user-friendly, requiring minimal sample preparation and training to operate [63,74]. The development of highly conductive, easily usable, and stable electrode materials might fulfill the possibility of rapid and ultrasensitive reading of sepsis biomarkers. Even though sepsis biomarkers are not always related directly to sepsis and are mostly caused by inflammation, their concurrent occurrence, especially in high concentrations, can be related to sepsis. Therefore, it is important to propose a device that allows for the simultaneous detection of different biomarkers at the same time, which will enable the differentiation of sepsis from other diseases associated with local inflammatory states. A detailed overview and comparison of electrochemical sensors will be described in the following sections.

### 5.4. Microfluidic Sensors 

Microfluidic sensors integrate with microfluidic chips to manipulate small volumes of fluids for the detection of pathogens or sepsis markers through various transducer mechanisms, including optical, mass spectrometry, and electrochemical methods. Microfluidic chips are designed for the rapid detection of sepsis biomarkers, such as the chip proposed by Zupančič et al. [75], which detects IL-6 with high sensitivity (63.1 pg mL^−1^), enabling early sepsis diagnosis. A definite advantage here is that a small amount of blood can be used, so the change in biomarker amount over time can be continuously monitored, which is very important in the diagnosis and treatment of sepsis. It should be borne in mind that when using small amounts of blood, the sensor must be extremely selective and accurate due to the large number of non-sepsis-related blood elements that can interfere with the measurement (Figure 2). In the case of microfluidic sepsis, the sensor can detect the volume of the analyte in the sample effectively, even at the microscale. However, microscale fabrication and operating equipment costs are expensive, as summarized in Table 1.

## 6. Strategies for Electrochemical Sepsis Sensors 

The detection of prognostic sepsis biomarkers (biomarkers that appear at an early stage of the disease) can substantially decrease mortality rates among sepsis patients. Conventional serological analysis or molecular approaches consume a lot of time, e.g., the blood culture method provides a result in more than 24–72 h. In electrochemical sensor techniques, quantification of sepsis biomarkers can be completed within a few minutes or hours. Typically, electrochemical sensors operate based on simple selector-specific interactions with the molecules of the target analyte to change the electric signal [74,76]. Here, signal changes can include voltage (potentiometric), current (amperometric, cyclic voltammetric, and differential pulse voltammetric), resistance (impedimetric), and conductivity (conductometric) methods [76,77]. The electrochemical sensor generally consists of three electrodes, namely working, counter, and reference electrodes. The analyte recognition moiety (e.g., antibody) is typically immobilized on the surface of a working electrode and allows for the selective attachment of a target analyte (antigen). After the analyte binds with the respective antibody, the corresponding electrical signal (current density or impedance change) indicates the analyte’s concentration. 

### Molecular Labels and Functionalization 

**Figure 6:**
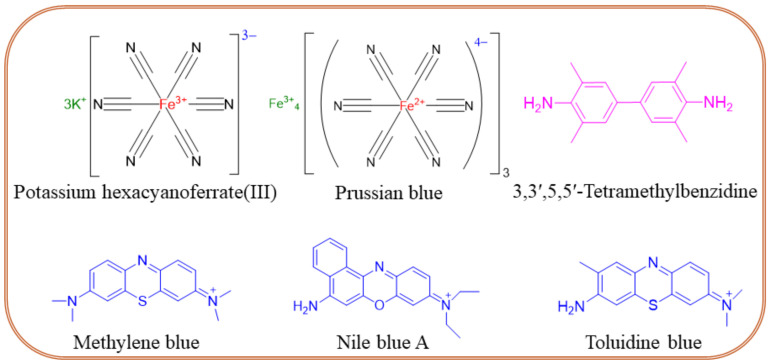
Chemical structures of redox couples frequently used in the design of sepsis sensors.

Molecular label-modified electrodes greatly influence the intensity of electrocatalytic currents after the addition of a target analyte, which correlates with the concentration of the unknown sample. The following molecular labels can be successfully used for the modification of biosensor electrodes. (i) Enzyme labels like horseradish peroxidase, alkaline phosphatase, glucose-6-phosphate dehydrogenase, glucose oxidase, etc. are interconnected with an electrochemical transducer system for the quantification of unknown samples. In this sense, enzymes must catalyze the enzymatic process rapidly and be highly stable on the electrode surface [78]. (ii) Oxidation–reduction peaks of nanoparticles are also used as labels for biomarker sensors. An example is a nanoparticle-grafted surface, which can be used to immobilize primary antibodies (Ab1). As a result of an antigen-antibody binding a peak current is reduced, and its decrease is related to the concentration of a biomarker. Qi et al. [79] reported a PCT sandwich-type sensor based on AgNPs, where Nile Blue A is adsorbed onto a metal-organic framework (UiO-67) for stability and biocompatibility and is used to label the secondary antibody (Ab2), which binds to PCT. The ratio of the NBA signal to the AgNPs signal is used to quantify the concentration of PCT, thereby enhancing accuracy. (iii) Quantum-modified electrodes can be also used as labels for electrochemical biosensors owing to their excellent photoelectrochemical properties. Quantum dot-modified electrodes exhibit a change in photocurrent response, which successfully translates to analyte quantification. For example, the photocurrent generated by Ag_2_S quantum dot-modified Bi_2_S_3_/ITO electrodes allows the detection of picogram levels of PCT [80]. (iv) Electrochemically active redox couple molecules serve as promising biomarker labels. The performance of electrochemical sensors primarily depends on the nature of a conductive substrate. The ideal substrate should exhibit high electric conductivity and allow for further functionalization through cross-linking agents like glutaraldehyde, 3-mercaptopropionic acid, ethanolamine, etc. Also, a high electron transfer rate between signal indicators of redox-active species is needed. Figure 6 shows the chemical structures of frequently used redox couples in sepsis sensors, namely potassium hexacyanoferrate (III) [Fe(CN)_6_]^3−/4−^, Prussian blue (PB), 3,3′,5,5′-tetramethylbenzidine (TMB), methylene blue (MB), Nile blue A, toluidine blue (TB), etc. The TMB, MB, Nile blue A, and TB form quinoid-type structures during the electrochemical oxidation process, offering a rapid electron transfer rate through ring electron delocalization. The redox probes are physically or covalently attached to antibodies in two ways. First, the redox probe is grafted on the primary Ab1 and monitors the decrease in the current intensity. This method seems facile for electrode fabrication though, and there is a chance for false results due to nonspecific adsorption. The second method involves a redox probe attached to the secondary antibody (Ab2) that enhances the peak current’s intensity. The second method is more interesting due to the lower chance of false results coming from specific attractions between antibodies and antigens. The use of TMB was reported by Zupančič et al. [75], where a low-noise sensor for multiplexed detection of sepsis markers was investigated. A BSA/rGOx/GA electrode was activated by EDC/NHS, then the antibody was immobilized. After the immobilization, the electrode was incubated with TMB, which precipitated on the surface where the enzyme was present. The local precipitation of TMB on the electrode surface ensured that the signal was confined, enhancing signal specificity, reducing background noise, and providing excellent sensitivity and selectivity. PBNC-AuNS-GO nanocomposite was proposed by Schuck et al. [81]. PB nanocubes improved the sensitivity and selectivity of the sensor by providing a stable and conductive matrix. They facilitated efficient electron transfer, enhancing the electrochemical signal corresponding to the presence of the target biomarkers. In addition, the presence of PB enhanced the sensor’s ability to detect hydrogen peroxide, which is crucial for the lactate detection mechanism. Other properties of the obtained sensor can be found in Table 2.

## 7. Electrochemical Detection of Sepsis Biomarkers

In this section, we discuss recent advancements in the electrochemical detection of the five most informative sepsis biomarkers (CRP, PCT, TNF-α, and IL-6), including the selection of electrode materials, electrode grafting strategies, antibody grafting methods, signal probe designs, and achievable limits of detections. 

### 7.1. Cyclic Voltammetry (CV)

Voltammetric techniques rely on the application of a voltage between the working and reference electrodes (ideal nonpolarizable electrode). The current, either an oxidation or reduction current, is measured as an output signal from the working electrode. If the redox couple is grafted on the working electrode, the electron transfer process falls on the surface-controlled kinetics. If the redox couple diffuses from the bulk solution to the electrode surface, it is defined in a diffusion-controlled manner. CV is a commonly used voltammetric technique and is effective in detecting the complicated electrochemical reaction on the electrode surface. In CV, potential is applied to the forward and reverse scans of the electroactive species. The nature of the voltammogram of electroactive species is identified as a reversible or irreversible reaction process. Typically, CV is used to observe the electroactive species oxidation–reduction peak potential and current intensity. 

CV was successfully used for the detection of PCT by employing a CdSeZnS quantum dot-modified electrode as an immunosensor electrode [82]. First, the indium tin oxide (ITO) surface was coated with 3-aminopropyltrimethoxysilane, and then cross-linked by GA and PCT-modified CdSeZnS quantum dots. The final system reached the low LOD value of 0.21 ng mL^−1^. Zupančič et al. [75] reported the application of a multiplexed gold electrode platform prepared using the photolithography technique for the detection of PCT. The conductive nanocomposite substrate was developed by grafting a mixture of amine-functionalized reduced graphene oxide, BSA, and glutaraldehyde (GA) on a gold multiplexed chip substrate, which was then incubated in PCT, CRP, pathogen-associated molecular patterns, and syndecan-1 capture probe. After coating the electrode with poly-HRP-streptavidin and TMB, the amount of TMB precipitation was measured using CV, and the area under the curve was used to quantify PCT. Unfortunately, the costs of photolithography and microfluidic cell assembly were significant and could be considered as limiting steps for commercial applicability. Another CV-based IL-6 sensor [83] was designed using a gold nanoflower-modified electrode. In this study, the change in the intensity of the [Fe(CN)_6_]^3−/4−^ redox probe was effectively monitored by the gold-integrated carbon fiber electrode, achieving a femtomolar level of IL-6 detection (1 fg mL^−1^ to 1 μg mL^−1^). 

### 7.2. Amperometry 

**Figure 7:**
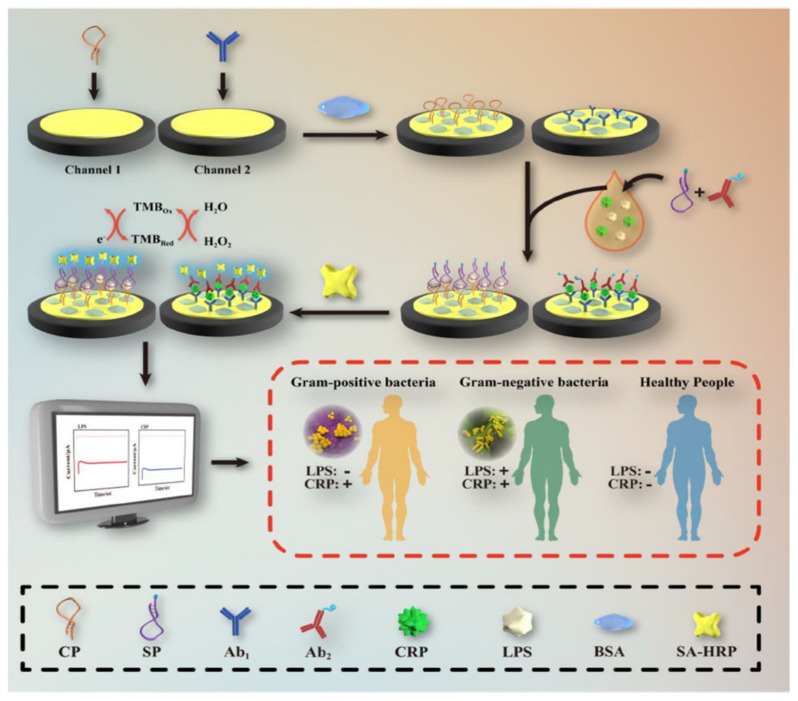
Systematic electrode design for CRP and lipopolysaccharide concomitant detection using the TMB and H_2_O_2_ electrocatalytic reduction method. Reprinted with permission from [85]. Copyright (2024) Elsevier.

In the amperometry technique, current changes are assessed as a function of time upon applying a constant voltage to the working electrode. The applied voltage reduces or oxidizes the electroactive species. Since the redox reaction occurs immediately on the working electrode surface, the current decreases with the electroactive species concentration. Amperometry is commonly used for diffusion-controlled kinetics processes and does not require the labeling of biomarkers. In the work of Zinggeler et al. [84], the UV illumination method was used for the immobilization of CRP antibodies through the process of crosslinking on an electrode substrate; the modified electrode achieved the amperometry technique using a wide range of CRP detection from 10 to 10,000 ng mL^−1^. Lu et al. [85] designed a CRP detection electrode using a dual-channel electrode assembled for the simultaneous lipopolysaccharide and CRP detection using a drop-casting approach on the gold electrode (Figure 7). In short, a CRP binding antibody was cast on the Au surface and coated with a premixed CRP sample containing a non-specific interaction inhibition composition of biotin-labeled CRP antibody and bovine serum albumin (BSA). Then, the electrode was covered with streptavidin-labeled horseradish peroxidase, which was able to chemically react with the redox mediator, TMB. The electrochemical reduction of TMB at +0.1 V was the source of a signal used to quantify CRP. 

**Figure 8:**
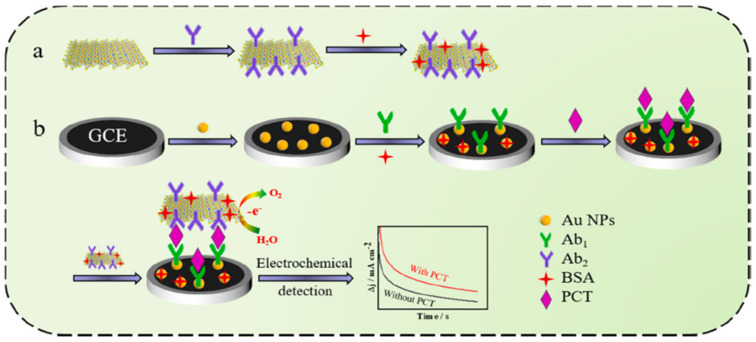
Schematic O_2_ evolution catalyst design with secondary Ab2 grafting and primary Ab1 electrode design for PCT detection: (**a**) preparation of electrocatalytic label; (**b**) fabrication of the immunosensor. Reprinted with permission from [86]. Copyright (2024) Elsevier.

An interesting option is to use a CoFe-oxyhydroxcide-Ab2 (secondary PCT antibody) oxygen evolution reaction (OER) catalyst, in which amperometry current density enhancement is related to the increase in PCT concentration [86]. This sandwich-type Ab1/PCT/An2-non-noble OER catalyst architecture (Figure 8) achieved the LOD of 0.33 pg mL^−1^. The photoelectrochemical method was also applied for the design of a PCT sensor, with the use of a Bi_2_S_3_/Ag_2_S catalyst for the production of photocurrent [80]. Owing to the specific attraction between the antigen and a modified electrode, photocurrent intensity was found to decrease owing to the electron transfer hindrance effect. The photoelectrochemical approach achieved an LOD of 0.18 ng mL^−1^. Despite the simplicity of this sensor, the photocurrent response was found to greatly depend on the thickness of the photocatalyst coating. Therefore, the proposed electrode design required plenty of time to optimize Bi_2_S_3_ coating thickness and control the size of Ag_2_S quantum dots. Ge et al. [87] designed an H_2_O_2_ reduction catalyst of AuPtCu/graphene-Co particles encapsulated by 3D nitrogen-doped carbon. PCT detection was accomplished using the decrease in the H_2_O_2_ reduction current measured owing to the interfacial electron transfer hindrance at the nanocatalyst active sites. The ternary metal dendrite structure with a carbon composite electrode attained a considerable linear range of PCT detection from 0.0001 to 100 ng mL^−1^.

**Figure 9:**
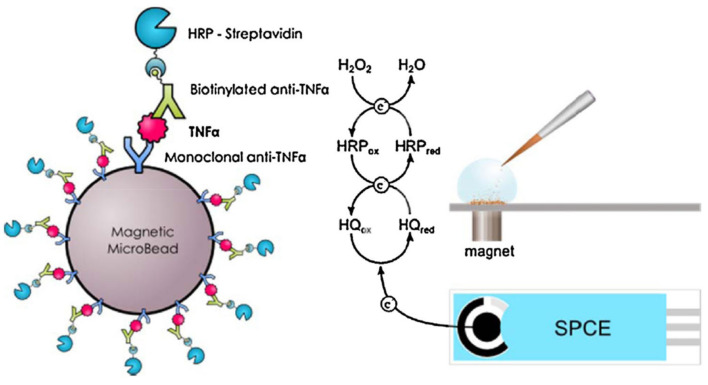
Magnetoimmunoassay electrode design for TNF-α through redox mediator reaction between the HQ and HRP. Reprinted with permission from [91]. Copyright (2024) Elsevier.

Fu et al. [88] reported CdS hollow cubes as a photoelectrode for photoelectrochemical TNF-α detection. NiCo_2_O_4_/Au@Apt was employed as a signal extinguisher, reducing the intensity of the photocurrent. In this approach, ultra-low LOD was achieved (0.63 fg mL^−1^). Though the photocurrent electrode was found to be excellent as a sensor, its applicability was limited by the use of toxic cadmium-based materials, which are unfavorable for health and the environment. The molecular imprinted (MIP) method was also explored for the design of TNF-α sensors. An efficient TNF-α sensor was fabricated using an electrode modified with zwitterionic phenyl phosphorylcholine and phenyl butyric acid [89]. In this study, Ab2-HRP secondary antibody was employed for the electrocatalytic reaction with ferrocenemethanol/hydrogen peroxide, which resulted in a wide range of TNF-α detection (0.01–500 ng mL^−1^). In this sensing system, the use of a redox mediator (ferrocenemethanol/H_2_O_2_) prevented the occurrence of false results. Tirado et al. [90] designed a probe that was able to simultaneously capture interleukin-1β and TNF-α on a dual screen-printed electrode. Here, biotinylated antibodies of IL-β1 and TNF-α were used for the electrocatalyst of poly-HRP-streptavidin-labeled moiety attraction. This protocol yielded a sensing range of 0.5–100 pg mL^−1^ for IL-1β and 1–200 pg mL^−1^ for TNF-α. The designed protocol delivered excellent simultaneous dual biomarker detection, with the only drawback being a lengthy procedure. Another study described the use of carboxylic acid functionalized magnetic bead (MGB) substrate for primary Ab1 modification, as presented in Figure 9 [91]. Biotinylated antibody and streptavidin–HRP design was used for the electrocatalytic current, which indicated the increase in TNF-α concentration (LOD of 2.0 pg mL^−1^).

### 7.3. Differential Pulse Voltammetry (DPV) 

In the DPV technique, a sequence of pulses with a fixed amplitude over a linear potential ramp is applied on the working electrode. Here, pulse potentials are superimposed. The current is collected at the beginning and end of the pulses, and the obtained values are subtracted. The pulse height is usually in the 10−100 mV range. The pulse duration and periods are commonly 50−100 ms and 1−2 s, respectively. This method gains a faradic current and eliminates the non-faradic or charging current. DPV was used as a method for the detection of CRP involving the silanization of triethoxysilylpropyl succinic anhydride on the surface of an indium tin oxide electrode and in the use of a new anhydride terminal group to react with the antibody’s amine group [92]. The detection was based on the redox signal of a ferrocenodimethanol redox couple, and its intensity changes were monitored through DPV measurement. This type of sensor was found to be sensitive, with a limit of detection of 0.34 μg mL^−1^. Another approach utilized nanocomposite-modified electrodes, particularly Au@CoFe/N-doped graphite carbon nanotube (N-GCNT) composite coated on the glassy carbon electrode, as well as CRP antibody-labeled MOF composite [93]. Though the proposed sensor demonstrated low LOD (0.167 ng mL^−1^), the nanoparticle synthesis and electrode assembly procedures were undoubtedly lengthy.

**Figure 10:**
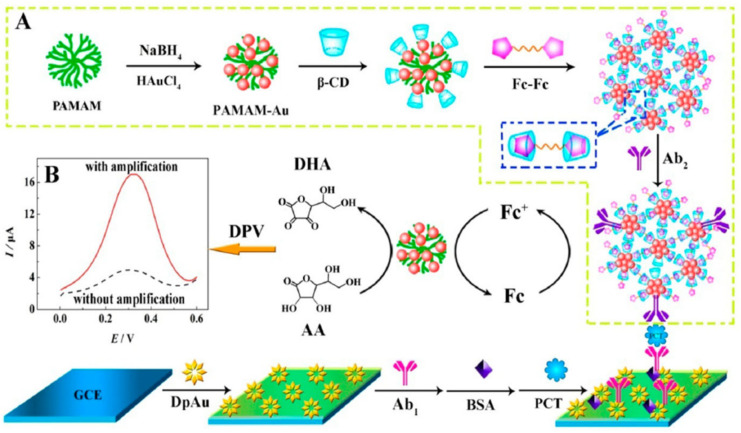
Schematic diagram of the ferrocene redox couple-mediated electrocatalytic PCT sensor: (**A**) preparation procedure of bioconjugates; (**B**) DPV curves with and without amplification Reprinted with permission from [94]. Copyright (2024) American Chemical Society.

Electrocatalytic signal amplification on the PCT detection electrode was achieved using a procedure involving: (1) the growth of gold nanoparticles on the surface of GCE followed by electrode modification with PCT antibody and BSA; and (2) the design of a nanonet using PAMAM-Au attached with β-cyclodextrins followed by N,N-bis(ferrocenoyl)diaminoethane amine/β-cyclodextrins-Ab2 signal amplifier assembly (Figure 10). This architecture allowed for the amplification of current intensity and provided a linear PCT sensing range (1.8–500 ng mL^−1^) but required an incubation time of 110 min [94]. A recent study [79] suggested the use of a dual signal change measurement for the detection of PCT. The dual signal sources are Ag nanoparticles decorated with a g-C_3_N_4_-Ab1 and UiO-67-Ab2 metal-organic framework surface modified with adsorbed Nile blue A (NBA). The current changes were measured using the DPV through the NBA current density increase (LOD 1.67 pg mL^−1^), and the Ag oxidation peak current decrease was related to the PCT concentration. 

Alternatively, Arya et al. [95] demonstrated a comb-shaped gold microarray electrode for the TNF-α sensor in an undiluted serum sample. A self-assembled monolayer of dithiobis (succinimidyl propionate) was used for the immobilization of the antibody, while a phosphate buffer-based starting block T20 acted as an antifouling reagent. The 4-aminophenyl phosphate was electrochemically oxidized, and the quinonimide concentration was monitored for TNF-α detection (linear range: 500 pg mL^−1^ to 100 ng mL^−^). An interesting electrode architecture was described by Hussain et al. [96], who studied a three-dimensional skyscraper (3D SS) electrode based on gold and polythiophene-2-carboxylicacid for grafting on the antibody. The ferrocenemethanol redox standard was used to detect TNF-α in a range from 60 to 1820 pg mL^−1^. In another study [97], gold interdigitated electrode array was employed for IL-6 detection in the cerebrospinal and serum samples, reaching LODs of 2.34 pg mL^−1^ and 11.83 pg mL^−1^, respectively. The interdigitated electrode was found to be an excellent IL-6 sensor in terms of its simple assembly, possibility of direct analysis, physiological medium, and simple operation. In another attempt [83], a nanoflower gold microstructure grown on the surface of carbon fiber was used for IL-6 detection, offering a low LOD of 0.056 fg mL^−1^. Vessella et al. [98] designed a V_2_CT_x_ MXene–Prussian blue/gold hybrid composite for the Ab2 signal amplifier and achieved a LOD of 0.5 pg mL^−1^. The proposed electrode design was applied for real-time sensing of IL-6 in breast cancer and epithelial cell medium. The V_2_CT_x_/PB/Au/Ab2 signal probe was found to exhibit better performance than that of Ti_3_C_2_T_x_/PB/Au/Ab2, which was justified by the hindrance issues. In another report [99], cobalt hexacyanoferrate redox couple was coated on a carboxyl functionalized MWCNTs/Au electrode and used for functionalization with IL-6 aptamer. The resultant peak current, associated with the reduction of cobalt, decreased with the increase in IL-6 concentration, allowing for sensitive IL-6 detection (LOD of 0.5–1000 pg mL^−1^). 

### 7.4. Square Wave Voltammetry (SWV) 

SWV is an amplitude differential method, in which a waveform is a symmetric square wave that is superimposed, and the staircase potential is applied at the working electrode. The current is collected twice during each cycle of the applied square wave, at the forward pulse end and reverse pulse end. The current is obtained by subtraction between the forward and reverse pulses. SWV greatly diminishes the charging current compared with the DPV, CV, and normal pulse voltammetry methods and achieves high sensitivity. Yang et al. [100] recently reported on CRP detection using the SWV technique. This group developed a peptide receptor graft on the surface of gold nanoparticles without the necessity to perform a coupling reaction. In short, black phosphorous was entrapped by dopamine through its self-polymerization process on the surface of gold nanoparticles. In this study, three different sequences of CRP-binding peptides were prepared and investigated. The black phosphorous and dopamine composite electrode yielded an excellent LOD of 0.7 ng mL^−1^. Finally, the proposed strategy was used for the analysis of blood samples collected from a Crohn’s disease patient. In another study [101], TNF-α aptamer was covalently bonded with methylene blue (MB) redox couple through the C3-terminal 6-disulfide linker. The SWV method was used for the measurement of MB redox current, reaching a decent LOD of 10 ng mL^−1^ within a short incubation time (15 min). MIP was realized with the use of ethylene glycol dimethacrylate, methyl methacrylate, and TNF-α antigen [102]. In this study, Fe_3_O_4_@SiO_2_/MoS_2_ base substrate for the MIP electrode offered picomolar detection and comparable selectivity without antibody immobilization. Qi et al. [103] reported the use of graphene oxide as a functional base material for the immobilization of IL-6 antibody and the use of Nile blue as a signal indicator. The graphene oxide composite electrode was successfully translated to living mice and was used to detect IL-6, with the sensitivity confirmed by an ELISA test study (LOD of 1 pg mL^−1^). Additional benefits of this sensor architecture included high reproducibility, easy storage, and low interference due to the high electron transfer rate of graphene oxide and the presence of easily accessible functional groups. 

### 7.5. Electrochemical Impedance Spectroscopy (EIS) 

In the EIS technique, a small-amplitude sinusoidal AC voltage, such as 2−10 mV, is applied to the working electrode, and the current response is measured. Here, the antibody is usually grafted on the working electrode surface, and the change in impedance and capacitance is assessed for the receptor’s concentration. EIS is a label-free, high-sensitivity, and economical method. Kim et al. [104] developed an EIS-based CRP sensor using a gold micro-gap electrode and were able to reduce the sample’s volume to 5 μL. The working electrode was prepared by the dissolution of porous rhodium nanoparticles in cysteamine and spreading them on a micro-gap electrode to achieve self-assembly. CRP-multifunctional DNA four-way junction/Ag^+^ was grafted by a thiol group, and the electron transfer rate was enhanced by intercalated silver ions. The developed electrode was able to detect CRP from 0.23 ng L^−1^ to 2.3 mg L^−1^. An approach to decrease the non-specific interactions on the electrode surface was proposed by Luo et al. [105], who modified the electrode by reacting four-armed polyethylene glycol epoxide with four-armed polyethylene glycol amine and used an amine-terminated polymer surface to graft CRP antibody using EDC/NHS chemistry. The resulted hydrophilic polyethylene glycol polymer greatly influenced nonspecific interfering moiety interactions on the electrode surface. This protocol employed a wide range of CRP detection from 500 pM to 50 000 pM. 

**Figure 11:**
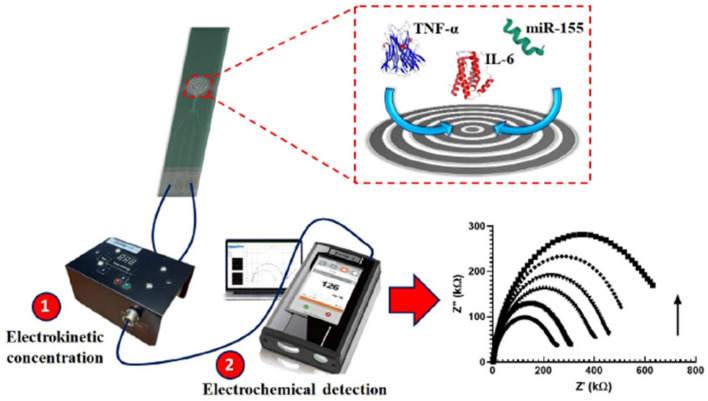
Photolithographic microelectrode design for the simultaneous TNF-α, IL-6, and microRNA-155 sensor electrode. Reprinted with permission from [111]. Copyright (2024) Elsevier.

A good example of a rapid and cheap PCT sensor was described by de Oliveira et al. [106], who coated the surface of an economical screen-printed array (at a cost of £1.14) with 4-aminobenzoic acid through diazotization and used the terminal carboxylic group for grafting PCT antibody through amide bond formation using EDC/NHS chemistry. An [Fe(CN)6]^3−/4−^ redox couple was used for the EIS signal change. Although the system was characterized by simple and cheap fabrication, the designed electrode offered a small range of PCT sensing (1–10 ng mL^−1^). On the other hand, a gold electrode modified with thiol-functionalized peptide was used for the impedimetric PCT detection, achieving a LOD of 12.5 ng mL^−1^ [107]. Unfortunately, this method did not exclude non-specific interactions, giving the possibility of false positive results. In another study [108], an interdigitated (laser engraved) graphene electrode with a customized pattern was fabricated and used for PCT sensing with a LOD of 0.36 pg mL^−1^. Despite their high sensitivity, engraved graphene electrodes are not economically beneficial, since the engraving process requires the purchase of high-quality and high-cost laser source accessories like lenses, motors, and mirrors. In another report [109], a single-layer graphene/Ag composite electrode was designed using a less expensive and easy electrode assembly method for PCT detection. Owing to the high electrical conductivity of a single graphene layer, the designed composite electrode showed excellent sensitivity (5.80 μA ng^−1^ mL cm^−2^) and a low LOD (0.55 ng mL^−1^). Another study [110] reported the design of an electrophoretic flower-like MoS_2_ and TNF-α coated ITO electrode, which achieved the lowest LOD detection (0.202 pg ml^−1^) in cancer patient blood samples. Ondevilla et al. [111] designed a point-of-care device for a series of TNF-α, IL-6, and microRNA-155 sensors using the photolithographic technique for microelectrode preparation (Figure 11). The aptamer-modified electrode revealed a direct relation between an increased impedance and increased concentration of the biomarker in mice. Also, the photolithography approach was used for the patterning of ITO working electrodes intended as TNF-α sensors [112]. Au/reduced graphene oxide composite was deposited on the patterned electrode using the potentiodynamic method, which conferred a low LOD (0.78 pg mL^−1^). Though Au/rGO/ITO achieved a commendable LOD, photolithography techniques are known to be expensive. In another report [113], a gold microelectrode was used as an Ab1 graft substrate, while Ab2 was functionalized with fluorescence moiety as a signal indicator. This electrode reached the high selectivity of TNF-α in the presence of IL-1 and IL-8 interring species and achieved a linear range from 1 to 15 pg mL^−1^. Salcedo et al. [114] reported the formation of a mercapto carboxyl self-assembled monolayer on the surface of a gold thin film array, which was used for the immobilization of an IL-6 aptamer capable of IL-6 sensing in the 10–10,000 pg mL^−1^ range.

Table 2 presents the details of the state-of-the-art electrochemical sensors, including electrode materials, electrode fabricating methods, sensing techniques, working concentration ranges, LODs, sensing times, sample volumes, and sample types.

## 8. Challenges in Electrochemical Sensing

Although electrochemical-based sensing methods have numerous advantages, they are not free of limitations. The most challenging issues that still need to be resolved are electrode fouling, the lack of quality antibody pairs with insufficient cross-linking reactivity, and limited sensitivity. Clinical samples have complex compositions and contain proteins, cells, biomolecules, and products of electrochemical reactions that can adsorb on an electrode’s surface. The unwanted fouling process results in the passivation of the electrode probe, which diminishes sensing sensitivity and specificity and increases noise levels. The majority of clinical samples, i.e., blood, saliva, cellular fluid, and plasma moieties, contain components that are non-specifically interrogated with the readers (antibodies) that considerably decrease the sensitivity. In addition, sepsis biomarker concentrations are often very low (0.0015%) compared with the background current of nonspecific interaction response. Therefore, even small changes in background signal noise, degradation, or current intensity might lead to false positive/negative results, severe method errors, and loss of specificity, which could eventually cause patient mortality. 

Numerous methods have been engineered to reduce electrode fouling and enhance sensitivity, with many of them using nanomaterials like gold nanoparticles, gold nanowires, carbon nanotubes, and amine-functionalized carbon materials. For instance, Li et al. [124] designed –NH_2_ group functionalized C60 particle-, carboxylic acid functionalized ferrocene-, and Pt nanoparticle-modified electrodes for PCT detection with a LOD of 6 pg mL^−1^. Sabaté del Río et al. [125] engineered a three-dimensional bovine serum albumin entangled gold nanowire nanocomposite electrode for an IL-6 sensor that revealed an excellent LOD (23 pg mL^−1^) and storage durability. A BSA@graphene-modified gold electrode achieved multiplexed and simultaneous diagnosis of sepsis biomarkers using PCT and CRP. Zha et al. [126] studied MXene@cellulose flexible membrane composite with excellent anti-biofouling properties. Nanocomposite architecture electrode assembly studies are a simple route to increasing the conductivity and suppressing the non-specific attraction of clinical sample moiety.

Organic molecule coatings on nanoparticles or carbon material surfaces efficiently reduce the foulants present in complex clinical biosamples. Recent studies explored the surface chemistry engineered for a particular matrix that selectively allowed the target analyte near the electrode surface (antibody); other interference was repelled. For instance, Jiang et al. [89] developed a small-chain zwitterionic moiety with poly(ethylene glycol) as an ant-biofouling reagent. This type of architecture allowed fouling over a prolonged time during biological fluid sample incubation. When the surface of polyaniline nanowires was functionalized with hyaluronic acid, the resulting structure exhibited suitable antifouling activities in the presence of both proteins and samples of human serum [127]. In contrast, the antifouling impact drastically decreased relative to the serum concentration and electrode soaking time in the sample. 

**Figure 12:**
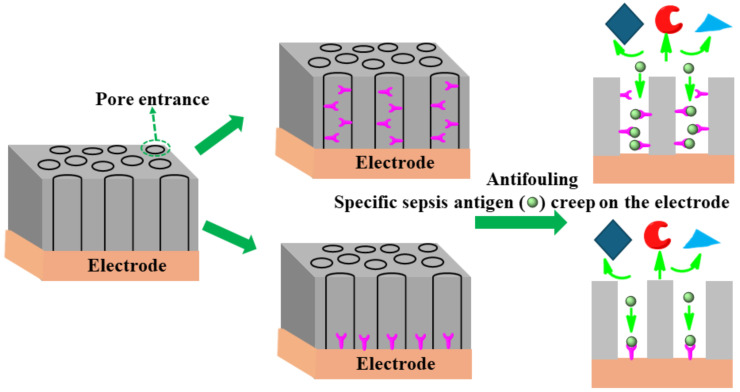
Schematic principles of several anti-fouling strategies based on nanoporous thin coatings.

The uniform size (shape) of nanopores coating the surface of an electrode plays a significant role in separating specific analytes/antibodies and offering high affinity of the antigen through chemical or electrostatic interactions [128]. The redox-active moiety can be tailored on a pore’s endogenous surface for analyte preconcentration using electrostatic/steric interactions. Having shown enhanced sensitivity, this type of nanoporous surface coating mitigates the fouling of clinical sample matrices containing unwanted biomolecules. The specific affinity of DNA or antigens creeps toward the pore owing to chemical/electrostatic interaction on the redox electrode surface. As an example of such an approach, Sun et al. [129] successfully designed an isoporous silica-micelleporous membrane electrode suitable for chloramphenicol sensing of whole-blood samples without experiencing biofouling. Harandizadeh et al. [130] reported polymer-based recessed nanodisk electrodes for electrochemical selective DNA sensors. The nanoporous coating approach might be fruitful in enhancing specificity, sensitivity, and antifouling architecture as a desirable approach to designing a sepsis biomarker sensor. Figure 12 shows the schematic principle of a nanopore design to achieve anti-fouling properties. 

The accurate diagnosis of sepsis biomarkers in terms of sensitivity, specificity, and low LOD is in high demand to reduce doctors’ burden on patient advancement. Because sepsis is a life-threatening condition, sensitive sensing of sepsis biomarkers is crucial for patient treatment. The major issue limiting the performance of presently available sepsis sensor electrode materials is low sensitivity due to whole-blood sample matrix fouling on the conductive electrode surface. This effect mitigates the rate of electron transfer and possibly increases the background (noise) signal, such as current capacity, and ultimately results in the loss of sensitivity and signal-to-noise values. Eventually, inferior sensor performance may result in false negative/positive results. 

Highly conductive nanomaterials, like carbon nanotubes, graphene, boron-doped diamond, gold nanoparticles, conducting polymers, and MXene-based materials, can greatly improve the sensitivity of biomarker readings. These conductive coatings, when deposited on an electrode surface, have been found to improve sensitivity, LOD, and comparable selectivity [131]. Recently, boron-doped diamond electrodes were extensively used for immunoassays owing to their promising wider potential window, low capacitive current, high stability, chemical inertness, and biocompatibility [132]. In this sense, disposable screen-printed electrodes (SPE) are extensively used for biosensor design and show high sensitivity with surface-charge-transfer functional groups, conductive nanocomposites, and synchronization of surface chemical modifications. Therefore, a highly conductive nanocomposite hybrid material tailored SPE electrode might be an appropriate substrate for antibody immobilization. 

## 9. Summary and Outlook

The imperative need for the early diagnosis of sepsis from patient blood samples is to provide reliable data without false negative/positive results to treat the patient before irreversible organ damage. Hence, it is important to ensure the design of a methodology for simple, bedside, and sensitive clinical whole-blood or human-serum sample analysis. The sensing devices must be designed to determine accurate readings even at low concentrations of biomarkers in the existing combinations of bioactive species. Additionally, concurrent multiple sepsis biomarker sensing, preferably using an array of electrode types is highly desirable. Traditional approaches seem highly sensitive; nonetheless, they are known as time-consuming techniques, thus they are completely unreliable for diagnostic requirements. Recent studies indicated the usefulness of electrochemical sensor leap research for ultrasensitive sepsis diagnosis, particularly utilizing advanced nanomaterials like functionalized MWCNTs, graphene, conducting polymers, gold nanostructures, MOF, and MXene-based materials. An electrode suitable for a biomarker sensor can be identified using two strategies, namely by a decrease in current intensity/increase in impedance as a result of capturing Ab1 after binding with antigen redox mediator, or by the increase in redox couple signal/decrease in impedance after the binding of Ab1 antigen with a secondary antibody (Ab2). Therefore, the stable grafting of an Ab1 probe on the conductive electrode surface is important to attain high sensitivity, a low LOD, and a stable signal. To resolve these issues, recent studies focused on Ab2 grafting with the conductive nanocomposite along with redox mediators, which could limit the occurrence of false results by amplifying the signals of interest. These studies employed dye-type redox couples as signal amplifiers on the Ab2 attachment composite; however, their stability is still a factor limiting their practical applicability. As a result of the judicious selection of electrode design, it is possible to detect sepsis biomarkers with unprecedented sensitivity, reaching LODs of 0.82 ag mL^−1^ for PCT, 0.167 ng mL^−1^ for CRP, 0.202 pg mL^−1^ for TNF-α, and 0.056 fg mL^−1^ for IL-6. Therefore, it is expected that electrically conductive, sensitive, target-specific, and non-specific interaction-restricted electrochemical sensors will soon replace the traditional analytical methods of diagnosis, allowing for the early diagnosis of sepsis and increasing the survival rates of patients.

## Figures and Tables

**Table 1:** Comparison of the working principles, advantages, and disadvantages of different types of sepsis sensors.

Type of Sensor	Working Principle	Pros	Cons	Ref.
Optical sensors	The bioreceptor is grafted on the surface, and interaction of the analyte with the receptor changes optical properties (resonant momentum, phase, polarization, etc.).	✓ high sensitivity ✓ ease of fabrication	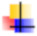 poor resolution with volume of the target analyte.	[65,66,67]
FET sensors	Biomarker binding on the bioreceptor-modified surface changes its potential. Variation in the electrostatic gating effect correlates with the concentration of the biomarker.	✓ high sensitivity ✓ mass production capability ✓ low-cost	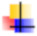 real samples with high ionic strength can cause false results.	[68,69]
Electrochemical sensor	Biochemical signal gives an electrical signal output. Biomarker binds to the bioreceptor-modified surface and changes electrode conductivity, which is measured as current, potential, or impedance.	✓ fast method ✓ low detection limit ✓ easy handling ✓ simple sample preparation ✓ high selectivity	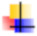 electrode fouling issue. 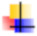 sensitivity issue due to insulating ability of bioreceptor and biomarkers. 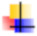 limited multiplexed biomarker sensor.	[70]
Microfluidic sensors	The microscale sample passes through micropumps, microchannels, microfluidic mixers, and valves. Analyte concentration is detected by various methods such as optical, electrochemical, mass spectrometry detection, nuclear magnetic resonance, magneto-resistive, and acoustical methods.	✓ fast method ✓ microscale volume sample effectively measured ✓ multiple biomarkers simultaneously detected ✓ small size of device	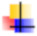 research needed on microfluidic and electrochemical transducer coupling. 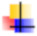 microscale device fabrication and operation are expensive. 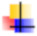 biomolecule/protein can block microchannels.	[71]

**Table 2:** Summary of the state-of-the-art electrochemical sensors used for the detection of sepsis biomarkers; limit of detection: LOD; electrochemical impedance spectroscopy: EIS; differential pulse voltammetry: DPV; cyclic voltammetry: CV; square wave voltammetry: SWV; C-reactive protein: CRP; procalcitonin: PCT; N-ethyl-N′-(3-(dimethylamino)propyl)carbodiimide/N-hydroxysuccinimide: EDC/NHS; Indium tin oxide: ITO; double-walled carbon nanotube: DWCNT; tumor necrosis factor-alpha: TNF-α; interleukin-6: IL-6; bovine serum albumin: BSA.

S. No	Electrode Material	Electrode Fabrication Method	Sensing Method/Technique	Linear Concentration Range	LOD	Sensing Time	Volume of The Sample	Sample Type	Ref.
**C-reactive protein**									
1	Gold electrode	CRP antibody drop casting method and antifouling reagent biotin-labeled BSA	Amperometry	0.1–20 μg mL^−1^	0.05 μg mL^−1^	75 min	3 μL	Plasma sample	[84]
2	Poly(N,N-dimethylacrylamide-stat-methacryloyloxy-benzophenone)/multiwallled carbon nanotube-COOH	Drop casting and photo crosslinker	Amperometry	10–10,000 ng mL^−1^	3.1 ng mL^−1^	10 min		Serum sample	[85]
3	Triethoxysilylpropyl succinic anhydride-modified indium tin oxide-coated glass electrodes	Silanization surface amide bond formation	DPV	1.0−100 μg mL^−1^	0.34 μg mL^−1^	30 min	-	-	[92]
4	Au@CoFe/N-doped graphene-carbon nanotube	EDC/NHS reagent for antibody grafting	DPV	0.5–200 ng mL^−1^	0.167 ng mL^−1^			Serum sample	[93]
5	CRP-Peptide-Au@black phosphorous/polydopamine	Drop casting	SWV	1−0.036 μg mL^−1^	0.7 ng mL^−1^			Crohn’s disease patient serum and plasma samples	[100]
6	Multifunctional DNA four-way junction-porous rhodium nanoparticle	CRP-DNA-4WJ self-assembly on electrode surface	EIS	1 pM–100 nM	0.349 pM (0.08 ng L^−1^)		5 μL	Serum sample	[104]
7	Poly(ethylene glycol)	Hydrophilic polyethylene glycol amine surface-grafted CRP antibody using EDC/NHS	EIS	500–50,000 pM	150 ± 10 pM	10 min	50 μL	Serum sample	[105]
Procalcitonin									
8	Ag-g-C_3_N_4_/GC UiO-67/NBA/Ab2	(1) Ab1 drop casting on Ag-g-C_3_N_4_/GC (2) Ab2 grafted on UiO-67/NBA using EDC/NHS	DPV	0.005–50 ng mL^−1^	1.67 pg mL^−1^	-	-	Serum sample	[79]
9	Bi_2_S_3_ and Ag_2_S quantum dot	ITO/Bi_2_S_3_/Ag_2_S-PCT-Ab/BSA	Photocurrent amperometry	0.0005–50 ng mL^–1^	0.18 ng mL^–1^	-	-	Serum sample	[80]
10	CdSeZnS quantum dot	CdSeZnS quantum dot-glutaraldehyde-ITO	CV	10–10,000 ng mL^−1^	0.21 ng mL^−1^	-	-	Serum sample	[82]
11	Au nanoparticles/CoFe-oxyhydroxcide	CoFe-(oxy)hydroxide-Ab2/PCT/BSA/Ab1/Au NPs/GC drop casting approach	Amperometry (OER method)	0.0005−100 ng mL^–1^	0.33 pg mL^–1^	~2 h	-	Serum sample	[86]
12	AuPtCu and Graphene-Co	Dendrite-like AuPtCu/G-Co/NCNBs/GC H_2_O_2_ reduction catalyst applied for PCT sensor	Amperometry	0.0001 to 100 ng mL^−1^	0.011 pg mL^−1^	50 min	-	-	[87]
13	Gold nanoparticles	Chemical and drop casting method	DPV	1.8–500 ng mL^−1^	0.36 pg mL^−1^	110 min		Serum sample	[94]
14	4-Aminobenzoic acid	4-Aminobenzoic acid surface graft with EDC/NHS reagent	EIS	1–10 ng mL^−1^	0.7 ng mL^−1^	-	-	Serum sample	[106]
15	Gold electrode	HS-Peptide (PCT T BP3) for PCT capture probe	EIS	0.0125–0.25 μg mL^−1^	12.5 ng mL^−1^	1 h	-	Serum sample	[107]
16	Laser-engraved graphene/Au	EDC/NHS reagent for PCT antibody graft	EIS	2.5–800 pg mL^–1^	0.36 pg mL^–1^	1 h	-	-	[108]
17	Ag@single layer graphene	Drop casting method for PCT Ab on Ag@SLG/ITO	EIS	4–25 ng mL	0.55 ng mL^−1^	1 h	-	-	[109]
18	Au@rGO	PCT-Ab/Au-RGO@Cellulose fiber	Amperometry	10–15,000 pg mL^−1^	10 pg mL^−1^	-	-	-	[115]
19	Au-rGO-cellulose fiber	CF/PEDOT:PSS-Au-rGO/PCT-Ab/BSA drop cast method	Amperometry	1000–6 × 10^6^ fg mL^−1^	280 fg mL^−1^	-	-	-	[116]
Tumor necrosis factor-alpha									
20	Apt_1_-CdS/ITONiCo_2_O_4_/Au@Apt_2_	Immersion and drop casting methods of Apt	Photocurrent (amperometry)	1 fg mL^−1^ to 1 ng mL^−1^	0.63 fg mL^−1^	50 min for TNF-α 100 min in the signal extinguisher	-	Serum sample	[88]
21	Phenyl phosphorylcholine/phenyl butyric acid	Zwitterionic species grafted by electro-diazotization, Ab1 grafted on ITO by classical EDC/NHS	Amperometry	0.01–500 ng mL^−1^	10 pg mL^−1^	1 h	40 μL	Whole blood sample	[89]
22	DWCNT	Drop casting and commercial Mix&Go, signal amplifier HRP with hydroquinone EC’ reaction	Amperometry	1–200 pg mL^−1^	0.85 pg mL^−1^	60 min		Serum sample	[90]
23	Microbead-COOH	Drop cast/EDC/NHS, HRP with hydroquinone EC’ reaction	Amperometry	15–405 pg mL^−1^	5.8 pg mL^−1^	1–3 h	-	Serum sample	[91]
24	Au microarray	Dithiobis(succinimidyl propionate) base self-assembly moiety for the antibody binding and blocking reagent engineering	DPV	0.5–100 ng mL^−1^	0.06 ng mL^−1^	20 min	50 μL	Undiluted serum	[95]
25	Gold/polythiophene-2-carboxylicacid	EDC/NHS coupling, ferrocenemethanol redox standard	DPV	60–1820 pg mL^−1^	44.5 pg mL^−1^	-	-	Fecal pellet sample	[96]
26	TNF-α-[HO(CH_2_)_6_-S-S-(CH_2_)_6_−]	Methylene blue grafted on TNF-α aptamer	SWV	0–100 ng mL^−1^	10 ng mL^−1^	15 min		Whole human blood	[101]
27	MoS_2_, Fe_3_O_4_@SiO_2_, and MIP polymer	Molecular imprinted method	SWV	0.01 pM–100 nM	0.01 pM	3 min	50 μL	-	[102]
28	MoS_2_	Drop casting method	EIS	0.01–200 pg mL^−1^	0.202pg mL^−1^	30 min	-	Cancer patient sample	[110]
29	Pt Microelectrode	Pt-S (Pt-bond with aptamer S group)	EIS	1–100,000 pg mL^−1^	-	5 min	-	Mice	[111]
30	Au/rGO/ITO composite electrode by photolithography	3-mercaptopropionic acid self-assembled layer surface antibody graft using NHS/EDC reagent	EIS	1−1000 pg mL^−1^	0.78 pg mL^−1^	3 h		Serum sample	[112]
31	Au microarray	Diazo grafting, EDC/NHS linkage	EIS	1–15 pg mL^−1^	-	-	--	Saliva	[113]
Interleukin-6									
32	PBNC(Prussian blue nanocubes)/AuNS/GO	Drop casting method	DPV	5−150 pg mL^−1^	0.141 pg mL^−1^	-	5 μL	Human serum	[81]
33	Au/CF(carbon fiber)	Electrodeposition	CV/DPV	1 fg mL^−1^ to 1 μg mL^−1^	0.056 fg mL^−1^	15 min	20 μL	Human serum	[83]
34	3-MPA/Au IDEA (gold interdigitated electrode arrays)	Self-assembly	DPV	1 pg mL^−1^1 μg mL^−1^	11.83 pg mL^−1^	30 min	-	Human cerebrospinal fluid and serum	[97]
35	V_2_CT_x_/PB/Au SSNPs-Ab	Drop casting	DPV	0.005–0.5 ng mL^−1^	0.5 pg/mL^−1^	24 h	-	Breast cancer cells	[98]
36	cMWCNTs(carboxylated multi-walled carbon nanotubes)/CoHCF (cobalt hexacyanoferrate)/AuNPs/GCE	Drop casting and self-assembly	DPV	0.5 pg mL^−1^–1000 pg mL^−1^	0.17 pg mL^−1^	-	-	Serum sample	[99]
37	Diazonium salt/SPEs	Electrodeposition	“Heat-transfer” method	5−1000 pg mL^−1^	3.37 pg mL^−1^	15 min	110 μL	Human plasma sample	[117]
38	HRP-anti-IL-6/nanogold/dendrimer/Au	Self-assembly	Conductometry	30 to 300 pg mL^−1^	10 pg mL^−1^	-	-	-	[118]
39	PC/AuNPs/4-MBA/IL-6 Ab	Performing the reaction in an incubator with EDC/NHS and modifying with antibodies	CV/DPV	100 pg mL^−1^–700 pg mL^−1^	3 pg mL^−1^	-	-	Serum sample	[119]
40	BSA/anti-IL-6/CSG/FTO (chitosan/genipin modified fluorine tin oxide electrode)	Drop casting	CV	0.05–1000 pg mL^−1^	0.03 pg mL^−1^	45 min	5 μL	Murine blood	[120]
41	HRP-Ab2-AuNP-PDOP@CNT	Self-assembly	Amperometry	4.0–8.0 × 10^2^ pg mL^−1^	1.0 pg mL^−1^	-	-	Serum sample	[121]
42	Ab2 –AgNP–TiP	Magnetic	Magnetic electrochemical	0.0005–10 ng mL^−1^	0.0001 ng mL^−1^	~45 min	-	Serum sample	[122]
43	SWCNT(single walled carbon nanotubes)/Au electrode	Electrodeposition and self-assembly	EIS	0.01–100 fg mL^−1^	of 0.01 fg mL^−1^	-	-	Serum sample	[123]

## Data Availability

Not applicable.

## References

[1] World Health Organization (2020). Global Report on the Epidemiology and Burden of Sepsis: Current Evidence, Identifying Gaps and Future Directions.

[2] Cecconi M., Evans L., Levy M., Rhodes A. (2018). Sepsis and septic shock. Lancet.

[3] World Health Organization. https://www.who.int/news-room/fact-sheets/detail/sepsis.

[4] Chicco D., Jurman G. (2020). Survival prediction of patients with sepsis from age, sex, and septic episode number alone. Sci. Rep..

[5] Labib A. (2019). Sepsis Care Pathway 2019. Qatar Med. J..

[6] Vergnano S., Sharland M., Kazembe P., Mwansambo C., Heath P.T. (2005). Neonatal sepsis: An international perspective. Arch. Dis. Child. Fetal Neonatal Ed..

[7] Calsavara A.J., Costa P.A., Nobre V., Teixeira A.L. (2021). Prevalence and risk factors for post-traumatic stress, anxiety, and depression in sepsis survivors after ICU discharge. Braz. J. Psychiatry.

[8] Gawlytta R., Brunkhorst F., Niemeyer H., Boettche M., Knaevelsrud C., Rosendahl J. (2020). Dyadic post-traumatic stress after intensive care: Case report of a sepsis patient and his wife. Intensive Crit. Care Nur..

[9] Manabe T., Heneka M.T. (2022). Cerebral dysfunctions caused by sepsis during ageing. Nat. Rev. Immunol..

[10] Angus D.C., van der Poll T. (2013). Severe sepsis and septic shock. N. Engl. J. Med..

[11] Arwyn-Jones J., Brent A.J. (2019). Sepsis. Surgery.

[12] Schwartz A., Gurman G., Cohen G., Gilutz H., Brill S., Schily M., Gurevitch B., Shoenfeld Y. (2002). Association between hypophosphatemia and cardiac arrhythmias in the early stages of sepsis. Eur. J. Intern. Med..

[13] Mayr F.B., Yende S., Angus D.C. (2014). Epidemiology of severe sepsis. Virulence.

[14] Hotchkiss R.S., Moldawer L.L., Opal S.M., Reinhart K., Turnbull I.R., Vincent J.-L. (2016). Sepsis and septic shock. Nat. Rev. Dis. Primers.

[15] Angus D.C., Linde-Zwirble W.T., Lidicker J., Clermont G., Carcillo J., Pinsky M.R. (2001). Epidemiology of severe sepsis in the United States: Analysis of incidence, outcome, and associated costs of care. Crit. Care Med..

[16] Bhat A., Asghar M., Raulia G., Mandal A.K. (2016). Improving multidisciplinary severe sepsis management using the Sepsis Six. Clin. Med..

[17] Kübler A., Adamik B., Ciszewicz-Adamiczka B., Ostrowska E. (2015). Severe sepsis in intensive care units in Poland--a point prevalence study in 2012 and 2013. Anaesthesiol Intensive Ther..

[18] Marik P.E. (2014). Don’t miss the diagnosis of sepsis!. Critical Care.

[19] Gupta E., Saxena J., Kumar S., Sharma U., Rastogi S., Srivastava V.K., Kaushik S., Jyoti A. (2023). Fast track diagnostic tools for clinical management of sepsis: Paradigm shift from conventional to advanced methods. Diagnostics.

[20] Hu B., Xiang H., Dong Y., Portner E., Peng Z., Kashani K. (2020). Timeline of sepsis bundle component completion and its association with septic shock outcomes. J. Crit. Care.

[21] bioMerieux Connection (2019). Diagnosis & Treating Sepsis: The Need for Speed. Online..

[22] Alba-Patiño A., Russell S.M., Borges M., Pazos-Pérez N., Álvarez-Puebla R.A., de la Rica R. (2020). Nanoparticle-based mobile biosensors for the rapid detection of sepsis biomarkers in whole blood. Nanoscale Adv..

[23] Magrini L., Gagliano G., Travaglino F., Vetrone F., Marino R., Cardelli P., Salerno G., Di Somma S. (2014). Comparison between white blood cell count, procalcitonin and C reactive protein as diagnostic and prognostic biomarkers of infection or sepsis in patients presenting to emergency department. Clin. Chem. Lab Med..

[24] Farkas J.D. (2020). The complete blood count to diagnose septic shock. J. Thorac. Dis..

[25] Agnello L., Giglio R.V., Bivona G., Scazzone C., Gambino C.M., Iacona A., Ciaccio A.M., Lo Sasso B., Ciaccio M. (2021). The value of a complete blood count (CBC) for sepsis diagnosis and prognosis. Diagnostics.

[26] Piagnerelli M., Boudjeltia K.Z., Vanhaeverbeek M., Vincent J.L., Hedenstierna G., Mancebo J., Brochard L., Pinsky M.R. (2009). Red blood cell rheology in sepsis. Applied Physiology in Intensive Care Medicine.

[27] Coelho F.R., Martins J.O. (2012). Diagnostic methods in sepsis: The need of speed. Rev. Assoc. Méd. Bras..

[28] Murono K., Hirano Y., Koyano S., Ito K., Fujieda K. (2003). Molecular comparison of bacterial isolates from blood with strains colonizing pharynx and intestine in immunocompromised patients with sepsis. J. Med. Microbiol..

[29] Ulrich S., Gottschalk C., Straubinger R.K., Schwaiger K., Dörfelt R. (2020). Acceleration of the identification of sepsis-inducing bacteria in cultures of dog and cat blood. J. Small Anim. Pract..

[30] Amornphimoltham P., Yuen P.S.T., Star R.A., Leelahavanichkul A. (2019). Gut leakage of fungal-derived inflammatory mediators: Part of a gut-liver-kidney axis in bacterial sepsis. Dig. Dis. Sci..

[31] Lima-Oliveira G., Volanski W., Lippi G., Picheth G., Guidi G.C. (2017). Pre-analytical phase management: A review of the procedures from patient preparation to laboratory analysis. Scand. J. Clin. Lab Investig..

[32] Siegel J.D., Rhinehart E., Jackson M., Chiarello L. (2007). 2007 Guideline for isolation precautions: Preventing transmission of infectious agents in health care settings. Am. J. Infect. Control.

[33] Creedon J.M.B., Davis H. (2012). Advanced Monitoring and Procedures for Small Animal Emergency and Critical Care.

[34] Bennett J.E., Dolin R., Blaser M.J. (2015). Mandell, Douglas, and Bennett’s Principles and Practice of Infectious Diseases.

[35] Bernini P., Bertini I., Luchinat C., Nincheri P., Staderini S., Turano P. (2011). Standard operating procedures for pre-analytical handling of blood and urine for metabolomic studies and biobanks. J. Biomol. NMR.

[36] Gong Y., Liang S., Zeng L., Ni Y., Zhou S., Yuan X. (2019). Effects of blood sample handling procedures on measurable interleukin 6 in plasma and serum. J. Clin. Lab. Anal..

[37] Lim S.H., Mix S., Xu Z., Taba B., Budvytiene I., Berliner A.N., Queralto N., Churi Y.S., Huang R.S., Eiden M. (2014). Colorimetric sensor array allows fast detection and simultaneous identification of sepsis-causing bacteria in spiked blood culture. J. Clin. Microbiol..

[38] Zhang L., Feng Y., Fu P. (2021). Blood purification for sepsis: An overview. Precis. Clin. Med..

[39] Girardot T., Schneider A., Rimmelé T. (2019). Blood purification techniques for sepsis and septic AKI. Semin. Nephrol..

[40] Biron B.M., Ayala A., Lomas-Neira J.L. (2015). Biomarkers for sepsis: What is and what might be?. Biomark. Insights.

[41] Pierrakos C., Velissaris D., Bisdorff M., Marshall J.C., Vincent J.-L. (2020). Biomarkers of sepsis: Time for a reappraisal. Crit. Care.

[42] Barichello T., Generoso J.S., Singer M., Dal-Pizzol F. (2022). Biomarkers for sepsis: More than just fever and leukocytosis-a narrative review. Crit. Care.

[43] Balayan S., Chauhan N., Chandra R., Kuchhal N.K., Jain U. (2020). Recent advances in developing biosensing based platforms for neonatal sepsis. Biosens. Bioelectron..

[44] Thangamuthu M., Santschi C., Martin J.F.O. (2018). Label-free electrochemical immunoassay for C-reactive protein. Biosensors.

[45] Ali N. (2020). Elevated level of C-reactive protein may be an early marker to predict risk for severity of COVID-19. J. Med. Virol..

[46] Sonuç Karaboğa M.N., Sezgintürk M.K. (2018). A novel silanization agent based single used biosensing system: Detection of C-reactive protein as a potential Alzheimer’s disease blood biomarker. J. Pharmaceut. Biomed. Anal..

[47] Kim K.-W., Kim B.-M., Moon H.-W., Lee S.-H., Kim H.-R. (2015). Role of C-reactive protein in osteoclastogenesis in rheumatoid arthritis. Arthritis Res. Ther..

[48] Moya F., Nieto A., R-Candela J.L. (1975). Calcitonin biosynthesis: Evidence for a precursor. Eur. J. Biochem..

[49] Agarwal R., Sharma K., Mehndiratta M., Mohta M., Srivastava H., Anthonio A.E. (2021). Role of repeat procalcitonin estimation at 48 hours for outcome in pregnancy associated sepsis: A prospective observational study. Obstet. Gynecol. Sci..

[50] Kiaheyrati N., Babaei A., Ranji R., Bahadoran E., Taheri S., Farokhpour Z. (2024). Cancer therapy with the viral and bacterial pathogens: The past enemies can be considered the present allies. Life Sci..

[51] Chu W.-M. (2013). Tumor necrosis factor. Cancer Lett..

[52] Georgescu A.M., Banescu C., Azamfirei R., Hutanu A., Moldovan V., Badea I., Voidazan S., Dobreanu M., Chirtes I.R., Azamfirei L. (2020). Evaluation of TNF-α genetic polymorphisms as predictors for sepsis susceptibility and progression. BMC Infect. Dis..

[53] Han Z., Li J., Yi X., Zhang T., Liao D., You J., Ai J. (2024). Diagnostic accuracy of interleukin-6 in multiple diseases: An umbrella review of meta-analyses. Heliyon.

[54] Habanjar O., Bingula R., Decombat C., Diab-Assaf M., Caldefie-Chezet F., Delort L. (2023). Crosstalk of inflammatory cytokines within the breast tumor microenvironment. Int. J. Mol. Sci..

[55] Van Damme J., Opdenakker G., Van Damme S., Struyf S. (2023). Antibodies as tools in cytokine discovery and usage for diagnosis and therapy of inflammatory diseases. Eur. Cytokine Netw..

[56] Durkin T.J., Barua B., Savagatrup S. (2021). Rapid detection of sepsis: Recent advances in biomarker sensing platforms. ACS Omega.

[57] Ge Y., Huang M., Yao Y.-M. (2021). The effect and regulatory mechanism of high mobility group box-1 protein on immune cells in inflammatory diseases. Cells.

[58] Alba-Patiño A., Vaquer A., Barón E., Russell S.M., Borges M., de la Rica R. (2022). Micro- and nanosensors for detecting blood pathogens and biomarkers at different points of sepsis care. Microchim. Acta.

[59] Bonini A., Carota A.G., Poma N., Vivaldi F.M., Biagini D., Bottai D., Lenzi A., Tavanti A., Di Francesco F., Lomonaco T. (2022). Emerging biosensing technologies towards early sepsis diagnosis and management. Biosensors.

[60] Browning L., Patel M.R., Horvath E.B., Tawara K., Jorcyk C.L. (2018). IL-6 and ovarian cancer: Inflammatory cytokines in promotion of metastasis. Cancer Manag. Res..

[61] Unver N., McAllister F. (2018). IL-6 family cytokines: Key inflammatory mediators as biomarkers and potential therapeutic targets. Cytokine Growth Factor Rev..

[62] Thévenot D.R., Toth K., Durst R.A., Wilson G.S. (2001). Electrochemical biosensors: Recommended definitions and classification1International Union of Pure and Applied Chemistry: Physical Chemistry Division, Commission I.7 (Biophysical Chemistry); Analytical Chemistry Division, Commission V.5 (Electroanalytical Chemistry).1. Biosens. Bioelectron..

[63] Kumar S., Tripathy S., Jyoti A., Singh S.G. (2019). Recent advances in biosensors for diagnosis and detection of sepsis: A comprehensive review. Biosens. Bioelectron..

[64] Sheini A. (2021). A point-of-care testing sensor based on fluorescent nanoclusters for rapid detection of septicemia in children. Sens. Actuators B Chem..

[65] Zhang K., Liu G., Goldys E.M. (2018). Robust immunosensing system based on biotin-streptavidin coupling for spatially localized femtogram mL−1 level detection of interleukin-6. Biosen. Bioelectron..

[66] Luo M., Xue X., Rao H., Wang H., Liu X., Zhou X., Xue Z., Lu X. (2020). Simply converting color signal readout into thermal signal readout for breaking the color resolution limitation of colorimetric sensor. Sens. Actuators B Chem..

[67] Estevez M.C., Alvarez M., Lechuga L.M. (2012). Integrated optical devices for lab-on-a-chip biosensing applications. Laser Photonics Rev..

[68] Vu C.-A., Chen W.-Y. (2019). Field-effect transistor biosensors for biomedical applications: Recent advances and future prospects. Sensors.

[69] Lee J.-O., So H.-M., Jeon E.-K., Chang H., Won K., Kim Y.H. (2008). Aptamers as molecular recognition elements for electrical nanobiosensors. Anal. Bioanal. Chem..

[70] Li S., Zhang H., Zhu M., Kuang Z., Li X., Xu F., Miao S., Zhang Z., Lou X., Li H. (2023). Electrochemical biosensors for whole blood analysis: Recent progress, Challenges, and Future Perspectives. Chem. Rev..

[71] Radhakrishnan S., Mathew M., Rout C.S. (2022). Microfluidic sensors based on two-dimensional materials for chemical and biological assessments. Mater. Adv..

[72] Chen F., Zhang Y., Wang M., Liu J., Hai W., Liu Y. (2024). Chitosan modified graphene field-effect transistor biosensor for ultrasensitive procalcitonin detection. Talanta.

[73] Schuck A., Kim H.E., Moreira J.K., Lora P.S., Kim Y.-S. (2021). A Graphene-based enzymatic biosensor using a common-gate field-effect transistor for L-lactic acid detection in blood plasma samples. Sensors.

[74] Russell C., Ward A.C., Vezza V., Hoskisson P., Alcorn D., Steenson D.P., Corrigan D.K. (2019). Development of a needle shaped microelectrode for electrochemical detection of the sepsis biomarker interleukin-6 (IL-6) in real time. Biosens. Bioelectron..

[75] Zupančič U., Jolly P., Estrela P., Moschou D., Ingber D.E. (2021). Graphene enabled low-noise surface chemistry for multiplexed sepsis biomarker detection in whole blood. Adv. Funct. Mater..

[76] Timilsina S.S., Jolly P., Durr N., Yafia M., Ingber D.E. (2021). Enabling multiplexed electrochemical detection of biomarkers with high sensitivity in complex biological samples. Accounts Chem. Res..

[77] Karimi-Maleh H., Orooji Y., Karimi F., Alizadeh M., Baghayeri M., Rouhi J., Tajik S., Beitollahi H., Agarwal S., Gupta V.K. (2021). A critical review on the use of potentiometric based biosensors for biomarkers detection. Biosens. Bioelectron..

[78] Koyappayil A., Lee M.-H. (2021). Ultrasensitive materials for electrochemical biosensor labels. Sensors.

[79] Yue Q., Li X., Xu X., Huang X., Cao D., Wei Q., Cao W. (2022). Ratiometric electrochemical immunoassay for procalcitonin based on dual signal probes: Ag NPs and Nile blue A. Microchim. Acta.

[80] Zhao G., Wang Y., Wang H., Bai G., Zhang N., Wang Y., Wei Q. (2023). Ultrasensitive photoelectrochemical immunoassay strategy based on Bi_2_S_3_/Ag_2_S for the detection of the inflammation marker procalcitonin. Biosensors.

[81] Schuck A., Kim H.E., Kang M., Kim Y.-S. (2022). Rapid detection of inflammation-related biomarkers using an electrochemical sensor modified with a PBNC-AuNS-GO-based nanocomposite. ACS Appl. Electron. Mater..

[82] Ghrera A.S. (2019). Quantum dot modified interface for electrochemical immunosensing of procalcitonin for the detection of urinary tract infection. Anal. Chim. Acta.

[83] Madhu S., Han J.H., Jeong C.W., Choi J. (2023). Sensitive electrochemical sensing platform based on Au nanoflower-integrated carbon fiber for detecting interleukin-6 in human serum. Anal. Chim. Acta.

[84] Zinggeler M., Schär S., Kurth F. (2022). Printed antifouling electrodes for biosensing applications. ACS Appl. Mater. Interfaces.

[85] Lu T.-C., Yang Y.-J., Zhong Y., Qiu Q.-Z., Chen Z.-H., Chen Y.-Z., Lei Y., Liu A.-L. (2024). Simultaneous detection of C-reactive protein and lipopolysaccharide based on a dual-channel electrochemical biosensor for rapid Gram-typing of bacterial sepsis. Biosens. Bioelectron..

[86] Zhao G., Yan Q., Wang B., Wang N., Duolihong B., Xia X. (2022). CoFe-(oxy)hydroxide as a novel electrocatalytic tag in immunosensing for ultra-sensitive detection of procalcitonin based on the oxygen evolution reaction. Bioelectrochemistry.

[87] Ge X.-Y., Zhang J.-X., Feng Y.-G., Wang A.-J., Mei L.-P., Feng J.-J. (2022). Label-free electrochemical biosensor for determination of procalcitonin based on graphene-wrapped Co nanoparticles encapsulated in carbon nanobrushes coupled with AuPtCu nanodendrites. Microchim. Acta.

[88] Fu Y., Fan B., Chang S., Guo D., Wang F., Pan Q. (2023). An ultrasensitive photoelectrochemical assay for tumor necrosis factor-alpha based on hollow CdS cubes as a signal generator and NiCo_2_O_4_-Au as a signal extinguisher. Analyst.

[89] Jiang C., Alam M.T., Silva S.M., Taufik S., Fan S., Gooding J.J. (2016). Unique sensing interface that allows the development of an electrochemical immunosensor for the detection of tumor necrosis factor α in whole blood. ACS Sens..

[90] Sánchez-Tirado E., Salvo C., González-Cortés A., Yáñez-Sedeño P., Langa F., Pingarrón J.M. (2017). Electrochemical immunosensor for simultaneous determination of interleukin-1 beta and tumor necrosis factor alpha in serum and saliva using dual screen printed electrodes modified with functionalized double–walled carbon nanotubes. Anal. Chim. Acta.

[91] Eletxigerra U., Martinez-Perdiguero J., Merino S., Villalonga R., Pingarrón J.M., Campuzano S. (2014). Amperometric magnetoimmunoassay for the direct detection of tumor necrosis factor alpha biomarker in human serum. Anal. Chim. Acta.

[92] Szot-Karpińska K., Kudła P., Orzeł U., Narajczyk M., Jönsson-Niedziółka M., Pałys B., Filipek S., Ebner A., Niedziółka-Jönsson J. (2023). Investigation of peptides for molecular recognition of C-reactive protein–theoretical and experimental studies. Anal. Chem..

[93] Li M., Xia X., Meng S., Ma Y., Yang T., Yang Y., Hu R. (2021). An electrochemical immunosensor coupling a bamboo-like carbon nanostructure substrate with toluidine blue-functionalized Cu(ii)-MOFs as signal probes for a C-reactive protein assay. RSC Adv..

[94] Shen W.-J., Zhuo Y., Chai Y.-Q., Yang Z.-H., Han J., Yuan R. (2015). Enzyme-free electrochemical immunosensor based on host–guest nanonets catalyzing amplification for procalcitonin detection. ACS Appl. Mater. Interfaces.

[95] Arya S.K., Estrela P. (2017). Electrochemical immunosensor for tumor necrosis factor-alpha detection in undiluted serum. Methods.

[96] Hussain K.K., Hopkins R., Yeoman M.S., Patel B.A. (2024). 3D printed skyscraper electrochemical biosensor for the detection of tumour necrosis factor alpha (TNFα) in faeces. Sens. Actuators B Chem..

[97] Oh C., Park B., Li C., Maldarelli C., Schaefer J.L., Datta-Chaudhuri T., Bohn P.W. (2021). Electrochemical immunosensing of interleukin-6 in human cerebrospinal fluid and human serum as an early biomarker for traumatic brain injury. ACS Meas. Sci. Au.

[98] Vessella T., Zhang H., Zhou Z., Cui F., Zhou H.S. (2023). In-situ synthesized V2CTx MXene-based immune tag for the electrochemical detection of Interleukin 6 (IL-6) from breast cancer cells. Biosens. Bioelectron..

[99] Li Y., Hua X., Wang J., Jin B. (2023). cMWCNT/CoHCF/AuNPs nanocomposites aptasensor for electrochemical detection of interleukin-6. Talanta Open.

[100] Yang H.J., Kim M.W., Raju C.V., Cho C.H., Park T.J., Park J.P. (2023). Highly sensitive and label-free electrochemical detection of C-reactive protein on a peptide receptor−gold nanoparticle−black phosphorous nanocomposite modified electrode. Biosens. Bioelectron..

[101] Liu Y., Zhou Q., Revzin A. (2013). An aptasensor for electrochemical detection of tumor necrosis factor in human blood. Analyst.

[102] Balayan S., Chauhan N., Kumar P., Chandra R., Jain U. (2022). Fabrication of a sensing platform for identification of tumor necrosis factor-alpha: A biomarker for neonatal sepsis. 3 Biotech.

[103] Qi M., Huang J., Wei H., Cao C., Feng S., Guo Q., Goldys E.M., Li R., Liu G. (2017). Graphene oxide thin film with dual function integrated into a nanosandwich device for in vivo monitoring of interleukin-6. ACS Appl. Mater. Interfaces.

[104] Kim J., Park J.-A., Yim G., Jang H., Kim T.-H., Sohn H., Lee T. (2021). Fabrication of an electrochemical biosensor composed of multi-functional Ag ion intercalated DNA four-way junctions/rhodium nanoplate heterolayer on a micro-gap for C-reactive protein detection in human serum. Analyst.

[105] Luo X., Xu Q., James T., Davis J.J. (2014). Redox and label-free array detection of protein markers in human serum. Anal. Chem..

[106] Roberto de Oliveira P., Crapnell R.D., Garcia-Miranda Ferrari A., Wuamprakhon P., Hurst N.J., Dempsey-Hibbert N.C., Sawangphruk M., Janegitz B.C., Banks C.E. (2023). Low-cost, facile droplet modification of screen-printed arrays for internally validated electrochemical detection of serum procalcitonin. Biosens. Bioelectron..

[107] Lim J.M., Ryu M.Y., Kim J.H., Cho C.H., Park T.J., Park J.P. (2017). An electrochemical biosensor for detection of the sepsis-related biomarker procalcitonin. RSC Adv..

[108] Amouzadeh Tabrizi M., Acedo P. (2022). An electrochemical immunosensor for the determination of procalcitonin using the gold-graphene interdigitated electrode. Biosensors.

[109] Selimoğlu F., Gür B., Ayhan M.E., Gür F., Kalita G., Tanemura M., Alma M.H. (2023). Silver nanoparticle doped graphene-based impedimetric biosensor towards sensitive detection of procalcitonin. Mater. Chem. Phys..

[110] Sri S., Chauhan D., Lakshmi G.B.V.S., Thakar A., Solanki P.R. (2022). MoS_2_ nanoflower based electrochemical biosensor for TNF alpha detection in cancer patients. Electrochim. Acta.

[111] Ondevilla N.A.P., Liu P.-W., Huang W.-T., Weng T.-P., Lee N.-Y., Ma S.-C., Huang J.-J., Wong T.-W., Chang H.-C. (2024). A point-of-care electrochemical biosensor for the rapid and sensitive detection of biomarkers in murine models with LPS-induced sepsis. Biosens. Bioelectron..

[112] Yagati A.K., Lee M.-H., Min J. (2018). Electrochemical immunosensor for highly sensitive and quantitative detection of tumor necrosis factor-α in human serum. Bioelectrochemistry.

[113] Longo A., Baraket A., Vatteroni M., Zine N., Baussells J., Fuoco R., Di Francesco F., Karanasiou G.S., Fotiadis D.I., Menciassi A. (2016). Highly sensitive electrochemical BioMEMS for TNF-α detection in human saliva: Heart failure. Procedia Eng..

[114] Sánchez-Salcedo R., Miranda-Castro R., de-los-Santos-Álvarez N., Lobo-Castañón M.J., Corrigan D.K. (2023). Comparing nanobody and aptamer-based capacitive sensing for detection of interleukin-6 (IL-6) at physiologically relevant levels. Anal. Bioanal. Chem..

[115] Gupta Y., Pandey C.M., Ghrera A.S. (2022). Reduced graphene oxide-gold nanoparticle nanohybrid modified cost-effective paper-based biosensor for procalcitonin detection. ChemistrySelect.

[116] Gupta Y., Pandey C.M., Ghrera A.S. (2022). Development of conducting cellulose paper for electrochemical sensing of procalcitonin. Microchim. Acta.

[117] Crapnell R.D., Jesadabundit W., García-Miranda Ferrari A., Dempsey-Hibbert N.C., Peeters M., Tridente A., Chailapakul O., Banks C.E. (2021). Toward the rapid diagnosis of sepsis: Detecting interleukin-6 in blood plasma using functionalized screen-printed electrodes with a thermal detection methodology. Anal. Chem..

[118] Liang K., Mu W., Huang M., Yu Z., Lai Q. (2006). Interdigitated conductometric immunosensor for determination of interleukin-6 in humans based on dendrimer G4 and colloidal gold modified composite film. Electroanalysis.

[119] Wang C., Xin D., Yue Q., Wan H., Li Q., Wang Y., Wu J. (2023). A novel electrochemical IL-6 sensor based on Au nanoparticles-modified platinum carbon electrode. Front. Bioeng. Biotechnol..

[120] de Matos Morawski F., Dias G.B.M., Sousa K.A.P., Formiga R., Spiller F., Parize A.L., Báfica A., Jost C.L. (2023). Chitosan/genipin modified electrode for voltammetric determination of interleukin-6 as a biomarker of sepsis. Int. J. Biol. Macromol..

[121] Wang G., Huang H., Zhang G., Zhang X., Fang B., Wang L. (2011). Dual amplification strategy for the fabrication of highly sensitive interleukin-6 amperometric immunosensor based on poly-dopamine. Langmuir.

[122] Peng J., Feng L.-N., Ren Z.-J., Jiang L.-P., Zhu J.-J. (2011). Synthesis of silver nanoparticle–hollow titanium phosphate sphere hybrid as a label for ultrasensitive electrochemical detection of human Interleukin-6. Small.

[123] Yang T., Wang S., Jin H., Bao W., Huang S., Wang J. (2013). An electrochemical impedance sensor for the label-free ultrasensitive detection of interleukin-6 antigen. Sen. Actuators B Chem..

[124] Li P., Zhang W., Zhou X., Zhang L. (2015). C60 carboxyfullerene-based functionalised nanohybrids as signal-amplifying tags for the ultrasensitive electrochemical detection of procalcitonin. Clin. Biochem..

[125] Sabaté del Río J., Henry O.Y.F., Jolly P., Ingber D.E. (2019). An antifouling coating that enables affinity-based electrochemical biosensing in complex biological fluids. Nat. Nanotechnol..

[126] Zha X.-J., Zhao X., Pu J.-H., Tang L.-S., Ke K., Bao R.-Y., Bai L., Liu Z.-Y., Yang M.-B., Yang W. (2019). Flexible anti-biofouling MXene/Cellulose fibrous membrane for sustainable solar-driven water purification. ACS Appl. Mater. Interfaces.

[127] Wang J., Hui N. (2018). A nonfouling voltammetric immunosensor for the carcinoembryonic antigen based on the use of polyaniline nanowires wrapped with hyaluronic acid. Microchim. Acta.

[128] Ito T., Nathani A. (2022). Electrochemical sensing at nanoporous film-coated electrodes. Electrochem. Sci. Adv..

[129] Sun Q., Yan F., Yao L., Su B. (2016). Anti-biofouling isoporous silica-micelle membrane enabling drug detection in human whole blood. Anal. Chem..

[130] Harandizadeh Z., Ito T. (2019). Block copolymer-derived recessed nanodisk-array electrodes as platforms for folding-based electrochemical DNA sensors. ChemElectroChem.

[131] Białobrzeska W., Ficek M., Dec B., Osella S., Trzaskowski B., Jaramillo-Botero A., Pierpaoli M., Rycewicz M., Dashkevich Y., Łęga T. (2022). Performance of electrochemical immunoassays for clinical diagnostics of SARS-CoV-2 based on selective nucleocapsid N protein detection: Boron-doped diamond, gold and glassy carbon evaluation. Biosens. Bioelectron..

[132] Siuzdak K., Niedziałkowski P., Sobaszek M., Łęga T., Sawczak M., Czaczyk E., Dziąbowska K., Ossowski T., Nidzworski D., Bogdanowicz R. (2019). Biomolecular influenza virus detection based on the electrochemical impedance spectroscopy using the nanocrystalline boron-doped diamond electrodes with covalently bound antibodies. Sens. Actuators B Chem..

